# Sugarcane *ScDREB2B-1* Confers Drought Stress Tolerance in Transgenic *Nicotiana benthamiana* by Regulating the ABA Signal, ROS Level and Stress-Related Gene Expression

**DOI:** 10.3390/ijms23179557

**Published:** 2022-08-23

**Authors:** Yufeng Chen, Zhu Li, Tingting Sun, Dongjiao Wang, Zhoutao Wang, Chang Zhang, Youxiong Que, Jinlong Guo, Liping Xu, Yachun Su

**Affiliations:** 1 Key Laboratory of Sugarcane Biology and Genetic Breeding, Ministry of Agriculture and Rural Affairs, Fujian Agriculture and Forestry University, Fuzhou 350002, China; 2 Key Laboratory of Sugarcane Biotechnology and Genetic Improvement (Guangxi), Ministry of Agriculture and Rural Affairs & Guangxi Key Laboratory of Sugarcane Genetic Improvement, Sugarcane Research Institute, Guangxi Academy of Agricultural Sciences, Nanning 530007, China

**Keywords:** sugarcane, DREB transcription factor, expression pattern analysis, self-activation activity, drought tolerance

## Abstract

The dehydration-responsive element-binding protein (DREB) is a subgroup member of the AP2/ERF family and actively participates in the response of plants to abiotic stress. Although *DREB* genes have been studied in a variety of plant species, there are few reports of *DREB* genes in sugarcane (*Saccharum* spp.). In this study, a novel full-length cDNA sequence of the *ScDREB2B-1* gene was cloned from the *Saccharum* hybrid ROC22, whose encoding protein contained only one AP2-conserved domain and was clustered into the DREB (A-2) subgroup. The diverse promoter elements in the *ScDREB2B-1* gene and the accumulated transcripts of its homologous gene (*SsAP2/ERF-107*) in *S. spontaneum* under drought stress suggest that the *ScDREB2B-1* gene may play a role in drought response. In addition, reverse transcription quantitative PCR analysis showed that the expression level of the *ScDREB2B-1* gene was upregulated in the root and leaf of ROC22 under polyethylene glycol, sodium chloride and abscisic acid (ABA) treatments. The yeast two-hybrid experiment demonstrated that ScDREB2B-1 had transcriptional self-activation activity. Compared with wild-type plants, the overexpression of the *ScDREB2B-1* gene improved the drought tolerance of the transgenic *Nicotiana benthamiana* by activating the ABA pathway to enhance the expression of the ABA-responsive gene (*NbNCED*) and ABA content, regulate the intracellular reactive oxygen species (ROS) level (enhance the transcripts of ROS synthase-related gene *NbRbohB* and the activities of catalase, peroxidase and superoxide dismutase) and increase the relative water content, proline content and expression level of osmotic stress-related genes (*NbERD* and *NbLEA*). Collectively, our data indicate that *ScDREB2B-1* is a stress-inducible and ABA-responsive transcription factor gene that responds to drought stress by regulating ABA signaling, ROS levels and stress-related gene expression. This study contributes to a better understanding of the biological function of *ScDREB2B-1*, which could serve as a foundation for future resistance breeding in sugarcane.

## 1. Introduction

Sugarcane (*Saccharum* spp.) is the most important sugar crop and accounts for 85.6% of sugar production in China [1]. Drought is one of the most adverse environmental factors, severely affecting crop growth, development and yield [2]. As reported, about 30% of sugarcane production depends on an adequate water supply [3]. The most effective strategy for improving the drought tolerance of sugarcane is breeding and cultivating resistant varieties. The method of traditional selection breeding is limited by the genetic diversity of the germplasm and the long cycle, but transgenic technology can help overcome these obstacles [4]. Transcription factors (TFs) are the main regulators of cell activity in plants, and they can regulate the expression of stress-responsive genes by specifically binding to the cis-acting elements in their promoters under different stress conditions [5]. The APETALA 2/ethylene-responsive element binding factor (AP2/ERF) is one of the largest TF families and can be divided into AP2 (APETALA 2), RAV (related to ABI3/VP1), ERF (ethylene response element binding protein) and Soloist subfamilies [6]. DREB (dehydration-responsive element-binding protein), a subtype of ERF TFs, contains only one AP2-conserved domain and is related to abiotic stress [6]. DREB is classified into six groups (A-1–A-6) according to the structure, phylogeny, chromosome position, conserved motif and sequence similarity of the AP2/ERF domain [7,8]. The 14th and 19th amino acid residues of the AP2-conserved domain in the DREB protein are mainly valine (V) and glutamate (E), respectively [9]. DREB can regulate the expression of stress response genes, not only through binding the DRE/CRT cis-elements that are involved in the abscisic acid (ABA)-independent signaling pathway, but also through the ABA-dependent signaling pathway [10,11]. The ABA-independent and ABA-dependent signal transduction pathways are the main abiotic stress response pathways in plants. Most DREB TFs are related to ABA-independent pathways, such as DREBlA/CBF3, DREBlB/CBFl and DREBlC/CBF2 [12,13]. However, a few ABA-responsive DREB TFs have also been found in plants, such as OsDREB1F, ZmDBF1, ZmDBF2 and AtCBF4 [14,15,16,17].

DREB IFs have been identified from a variety of plant species, including *Oryza sativa* [18,19,20], *Arabidopsis thaliana* [7,21,22,23], *Triticum aestivum* [15], *Sorghum bicolor* [24], *Solanum lycopersicum* [25], *Glycine max* [26], *Malus pumila* [27], *Camellia sinensis* [28] and *Leymus chinensis* [21]. They are the most important regulons in genetic engineering for improving the stress tolerance of plants. In transgenic *A. thaliana* plants, the overexpression of *DREB1A* enhanced the expression level of stress-inducible downstream genes (such as *rd29A*, *cor15a*, *cor15b*, *kin1*, *kin2*, *erd10*, *rd17* and *AtGoIS3*) and improved the tolerance of *Arabidopsis* to cold, drought and high salt stress [22]. After overexpressing the *O. sativa OsDREB2B* gene in *A. thaliana*, the expression level of *DREB2A* target genes (*RD29A*, *RD29B*, *LEA14*, *HsfA3* and *Hsp70*) was increased, and the tolerance of transgenic plants to drought and heat stress was enhanced [29]. The overexpression of the *O. sativa OsDREB2A* gene in *G. max* significantly increased the transcription level of *GmDREB6*, *GmP5CS*, *GmERF3* and *GmERF7* genes and the accumulations of soluble sugar, free proline and other permeable substances, thus improving the tolerance of *G. max* to salt stress [19].

At present, there are few reports on DREB TFs in sugarcane and related wild species. Chanprame et al. [30] analyzed the sequence of the DREB A-2 subgroup gene *ScDREB2* in three sugarcane genotypes (wild, commercial cultivar and interspecific hybrid), and they found that the expression level of this gene increased under salt stress. Malhotra et al. [31] transformed the synthetic *AtDREB1A* gene into the commercial sugarcane variety ‘CoJ 83′, and after drought, salt and cold treatments, the expression level of *AtDREB1A* in transgenic plants was 6–18-fold higher than in wild-type (WT) plants. The overexpression of *Arabidopsis AtDREB2A CA* gene in sugarcane variety ‘RB855156′ increased its sucrose content, germination rate and drought tolerance without causing biomass loss [23]. Li [32] found that the sugarcane DREB (A-2) subgroup gene *SoDREB2* played a positive regulatory role in the drought resistance of transgenic *Nicotiana tabacum*. The expression level of *ErDREB1A* in a sugarcane wild species of *Erianthus fulvus* ‘Kunming99-1′ increased gradually with the extension of the low-temperature stress time [33]. *EaDREB2B*, a *DREB2B* gene isolated from the sugarcane-related species *E. arundinaceus* Retz., has been shown to improve the drought resistance of sugarcane plants [34,35,36]. Augustine et al. [37] found that the overexpression of another *EaDREB2* gene from *E. arundinaceus* ‘IK76-81′ with a pea DNA helicase gene (*PDH45*) enhanced the drought resistance and salt tolerance of *Saccharum* spp. hybrid Co 86032.

Because the genes encoding DREB are a multigene family, identifying and validating critical *DREB* genes that are involved in adversity stress responses are essential for the development of molecular breeding for stress resistance in sugarcane. In this study, a novel *ScDREB2B-1* gene from the *Saccharum* hybrid ROC22 (the prevalent sugarcane cultivar in mainland China) was cloned and identified. Its gene sequence, promoter elements, transcriptional self-activation activity, expression level under salt, drought and ABA stress were analyzed. In addition, the overexpression vector of the *ScDREB2B-1* gene was constructed and transformed into *N. benthamiana*. The drought resistance function of the *ScDREB2B-1* gene was verified by observing the phenotype, physiology, biochemistry change and expression level of stress resistance-related genes in transgenic *N. benthamiana* plants. This study was conducted with the aim of revealing the physiological and molecular basis of the *ScDREB2B-1* gene in improving drought tolerance and providing a candidate gene for the subsequent genetic improvement of sugarcane stress resistance.

## 2. Results

### 2.1. ScDREB2B-1 Belonged to the A-2 Group of the DREB Transcription Factor Family

**Figure 1:**
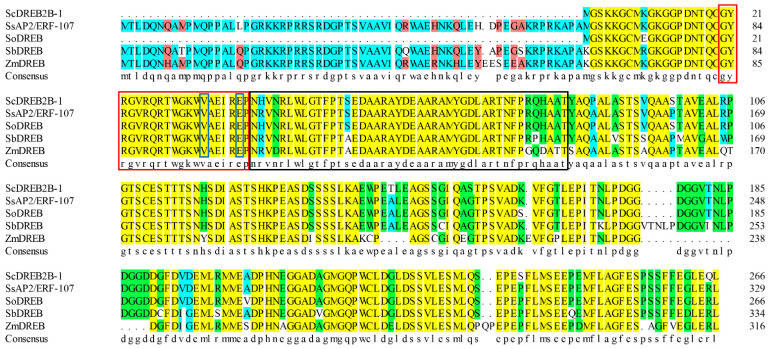
Amino acid sequence alignment of the ScDREB2B-1 protein and its four homologous proteins. Red box: YRG conserved element; black box: RAYD conserved element; blue box: the 14th valine (V14) and 19th glutamic acid (E19) residues in the AP2 conservative domain. SsAP2/ERF-107: *Saccharum spontaneum* DREB protein; SoDREB: *S. officinarum* DREB protein; SbDREB: *Sorghum bicolor* DREB protein; ZmDREB: *Zea mays* DREB protein. The yellow background showed identical residues; the green background and the blue background were blocks of similar residues; the red background depicted the weakly similar residues; the white background represented non-similar residues.

**Figure 2:**
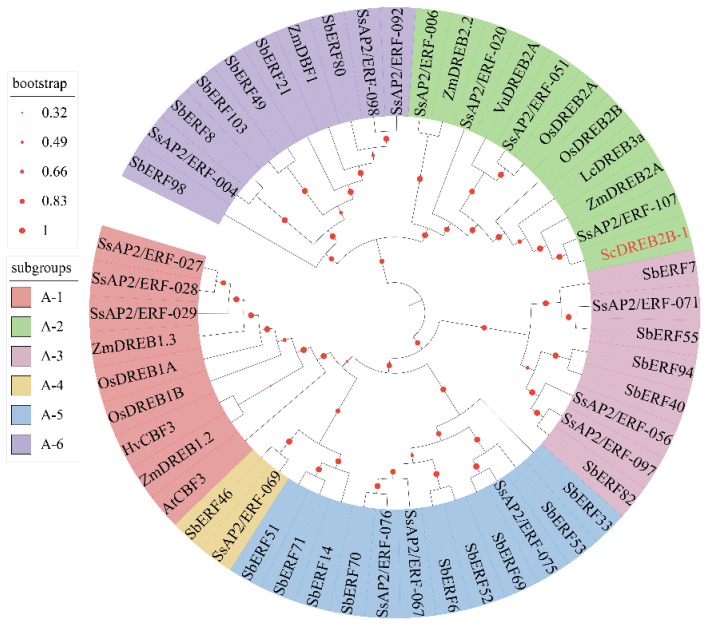
Phylogenetic tree analysis of ScDREB2B-1 protein in sugarcane and typical DREB (A-1-A-6) proteins in other plant species. The red font was the ScDREB2B-1 protein. Os, Zm, Sb, Ss, Lc, Vu, At and Hv represented *Oryza sativa*, *Zea mays*, *Sorghum bicolor*, *Saccharum spontaneum*, *Leymus chinensis*, *Vigna unguiculata*, *Arabidopsis thaliana* and *Hordeum vulgare*, respectively.

A full-length cDNA sequence of *ScDREB2B-1* (GenBank accession no. OM001106) was cloned from the elite sugarcane cultivar ROC22 using reverse transcription PCR (RT-PCR). *ScDREB2B-1* contained an open reading frame of 804 bp in length and encoded 267 amino acids. The isoelectric point and molecular weight of the ScDREB2B-1 protein were 5.11 and 65.28 kDa, respectively. The similarities of the amino acid sequence between ScDREB2B-1 and the DREB proteins from *S. spontaneum* (SsAP2/ERF-107, Sspon.07G0010180-1A), *S. officinarum* (AIN44351.1), *S. bicolor* (SbDREB, XP_021302434.1) and *Z. mays* (ZmDREB, PWZ09406.1) were 78.79, 97.38, 71.94 and 64.67%, respectively. Multiple sequence alignment analysis showed that the complete AP2 domain of these five proteins was composed of YRG (red box) and RAYD (black box) conserved elements, and its 14th and 19th amino acids (blue boxes) were valine (V) and glutamic acid (E), respectively, which was consistent with the sequence characteristics of the DREB protein (Figure 1). Phylogenetic tree analysis showed that ScDREB2B-1 belonged to the A-2 group of the DREB transcription factor family (Figure 2). In addition, ScDREB2B-1 was clustered in the same evolutionary branch as the reported *Zea mays* ZmDREB2A protein (Figure 2), suggesting similar functions.

### 2.2. Transcripts of ScDREB2B-1 Could Be Induced by Sodium Chloride, Polyethylene Glycol and Abscisic Acid Stress

**Figure 3:**
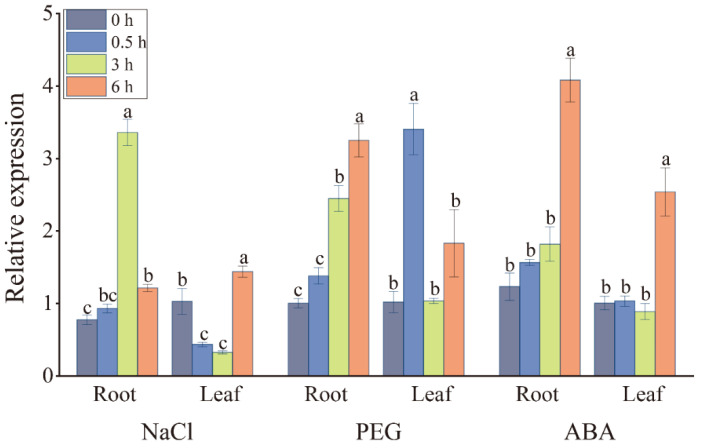
Expression of the *ScDREB2B-1* gene in sugarcane hybrid ROC22 under sodium chloride (NaCl), polyethylene glycol (PEG) 8000 and abscisic acid (ABA) treatments by RT-qPCR analysis. Quantitative data were normalized by the expression level of double references of *CAC* (clathrin adaptor complex) and *CUL* (cullin) genes. All expression data points were means ± standard error (*n* = 3). Significant differences were found between different letter substitutes on the column calculated by Duncan’s new multiple range test (*p*-value < 0.05).

The expression level of the *ScDREB2B-1* gene in the leaf and root of ROC22 under sodium chloride (NaCl), polyethylene glycol (PEG) and ABA treatments was detected by reverse transcription quantitative PCR (RT-qPCR) (Figure 3). After 250 mM NaCl stress, the expression level of *ScDREB2B-1* in the roots of ROC22 increased significantly at 3–6 h, which was 4.35 and 1.57 times that of the control (0 h), respectively. At 0.5–3 h, the expression level of *ScDREB2B-1* decreased significantly in ROC22 leaves, but increased significantly and reached a peak at 6 h, which was 1.40 times that of the control. Under the 20% PEG 8000 treatment, the expression level of *ScDREB2B-1* in the ROC22 roots increased significantly at 3 h and reached its peak at 6 h, which was 3.25 and 3.24 times that of the control. In the ROC22 leaf, the expression level of *ScDREB2B-1* increased significantly at 0.5 h, which was 3.34 times that of the control, and returned to the control level from 3 to 6 h. After applying the exogenous hormone ABA, the expression level of *ScDREB2B-1* in the roots and leaves of ROC22 was maintained at the control level at 0.5–3 h, increased significantly and reached a peak at 6 h, which were 3.31 and 2.53 times that of the control, respectively. These findings reveal that *ScDREB2B-1* is a stress-inducible transcription factor gene.

### 2.3. The Promoter of ScDREB2B-1 Was Rich in Cis-Acting Elements Related to Stress and the Expression of Its Homologous Gene in S. spontaneum Could Also Be Induced by Drought Stress

**Figure 4:**
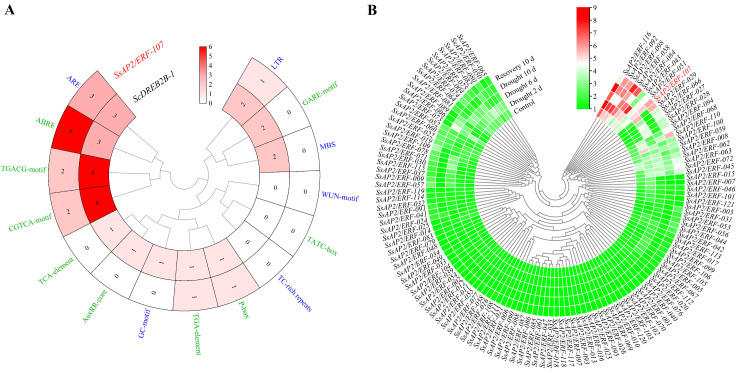
Promoter elements analysis of the *ScDREB2B-1* gene and expression profiles of the *Saccharum spontaneum* SsAP2/ERF gene family under drought stress. (**A**) Promoter elements analysis between *ScDREB2B-1* and its homologous gene *SsAP2/ERF-107* in *S. spontaneum*. Green and blue fonts represented hormone responsive elements and environmental stress-related elements, respectively. (**B**) Expression profiles of *S. spontaneum* SsAP2/ERF gene family under drought stress. The red font was *SsAP2/ERF-107*, which was a member of the ERF subfamily and a homologous gene of *ScDREB2B-1*. The expression heat map of *SsAP2/ERF* was constructed by TBtools with the transcript level transformed by log_2_ (FPKM + 1). FPKM mean fragments per kb per million.

To further understand the function of the *ScDREB2B-1* gene, its promoter sequence was cloned from ROC22 genome DNA based on the putative promoter sequence of the *SsAP2/ERF-107* gene (Figure S1). The prediction results showed that the promoter of *SsAP2/ERF-107* contained hormone response elements (ABRE, CGTCA-motif, P-box, TGACG-motif and TGA-element) and environmental and stress-related elements (ARE and LTR). The promoter elements in the *ScDREB2B-1* were more abundant than those in the *SsAP2/ERF-107* gene. The *ScDREB2B-1* gene also contained AuxRR-core, GARE-motif, TCA-element, GC-motif and MBS elements (Figure 4A). It is speculated that *ScDREB2B-1* is involved in the response to ABA, methyl jasmonate (MeJA), auxin, anaerobic induction and low temperature and drought stress. In addition, based on the transcriptome data, the expression profiles of some *S. spontaneum* SsAP2/ERF gene family members responded positively to drought stress (Figure 4B). Among them, the expression level of the homologous gene (*SsAP2/ERF-107*) of *ScDREB2B-1* increased gradually from 0 d to recovery at 10 d (Figure 4B). It is speculated that the *ScDREB2B-1* gene may play an important role in drought stress.

### 2.4. ScDREB2B-1 Protein Has Transcriptional Self-Activation Activity

**Figure 5:**
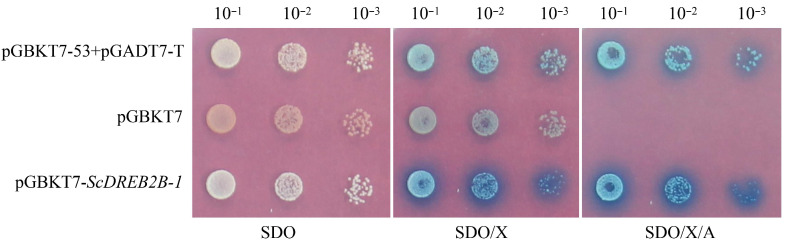
The transcriptional self-activation activity of ScDREB2B-1 protein in yeast. SDO: media without tryptophan; SDO/X: media without tryptophan but containing Trp/X-α-Gal dye; SDO/X/A: media without tryptophan but with Trp/X-α-Gal dye and aureobasidin A. pGBKT7-53+pGADT7-T: positive control; pGBKT7: negative control; pGBKT7-*ScDREB2B-1*: yeast transformant with pGBKT7-*ScDREB2B-1*.

Y2H Gold cells that transformed with the recombinant vectors of pGBKT7-53+pGADT7-T (positive control), pGBKT7 (negative control) and pGBKT7-*ScDREB2B-1* were all white on SDO media, indicating that these transformations were successful. The Y2H Gold transformants with pGBKT7-*ScDREB2B-1* were blue on SDO/X and SDO/X/A media, indicating that the ScDREB2B-1 protein had self-activation activity (Figure 5).

### 2.5. Overexpression of ScDREB2B-1 Improved Drought Tolerance of Transgenic N. benthamiana by Increasing Proline Content and Water-Retention Ability

**Figure 6:**
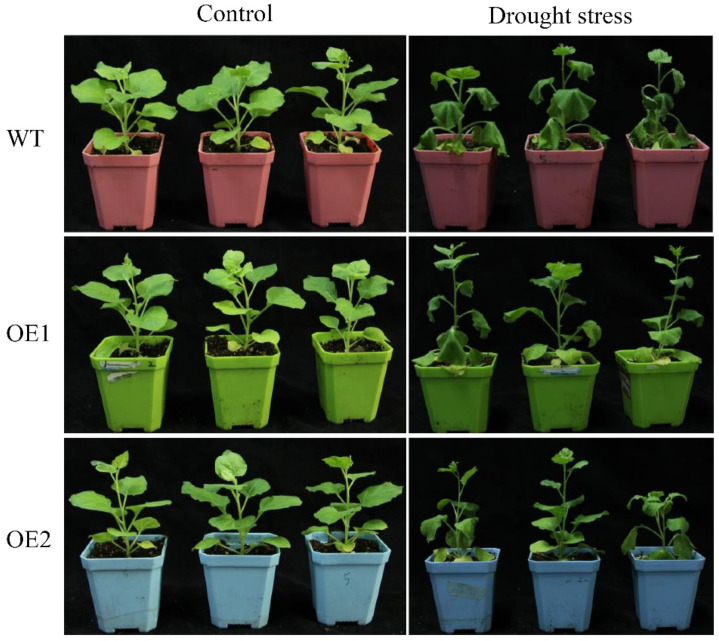
Phenotypes of transgenic *Nicotiana benthamiana* plants overexpressed sugarcane *ScDREB2B-1* under drought stress for 2 d. WT: wild-type *N. benthamiana*. OE1 and OE2: different transgenic *N. benthamiana* lines overexpressed *ScDREB2B-1*.

**Figure 7:**
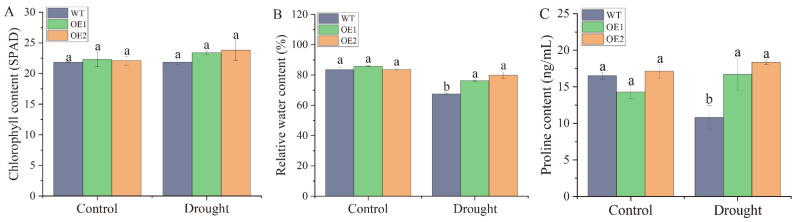
Contents of chlorophyll, relative water and proline in transgenic *Nicotiana benthamiana* plants overexpressed the *ScDREB2B-1* gene under drought stress for 2 d. (**A**) Chlorophyll content. (**B**) Relative water content. (**C**) Proline content. WT: wild-type *N. benthamiana*. OE1 and OE2: different transgenic *N. benthamiana* lines overexpressed *ScDREB2B-1*. All data points were means ± standard error (*n* = 3). Significant differences were found between different letter substitutes on the column calculated by Duncan’s new multiple range test (*p*-value < 0.05).

After stable genetic transformation, two homozygous transgenic lines, OE1 and OE2, were randomly selected from the T_3_ generation of *N. benthamiana* seeds overexpressing the *ScDREB2B-1* gene. Under drought stress for 2 d, the wild-type (WT) plants were wilted, and the degree of withering was much more severe than in the transgenic lines (Figure 6). There was no significant difference in chlorophyll content between the WT and transgenic lines under drought treatment (Figure 7A). However, the relative water content (RWC) of transgenic lines OE1 and OE2 was 1.13 and 1.18 times higher, respectively, than those of the WT plants (Figure 7B). Under normal conditions, there was no significant difference in proline content between the WT and transgenic lines. After 2 days of drought treatment, the proline content of OE1 and OE2 was significantly increased, which was 1.54 and 1.70 times that of the WT plants, respectively (Figure 7C). These results indicate that the overexpression of *ScDREB2B-1* enhanced the drought tolerance of *N. benthamiana* plants to a certain extent by increasing the proline content and water-retention ability.

### 2.6. Overexpression of ScDREB2B-1 Improved the Tolerance of Transgenic N. benthamiana to Drought Stress by Regulating the ABA Signal

**Figure 8:**
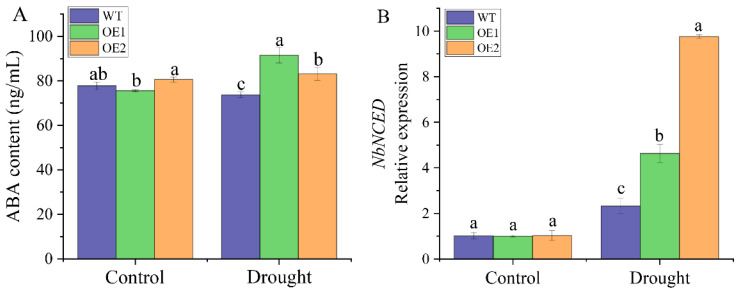
Determination of abscisic acid (ABA) content and expression of its response gene *NbNCED* in transgenic *Nicotiana benthamiana* plants overexpressed the *ScDREB2B-1* gene under drought stress for 2 d. (**A**) ABA content. (**B**) Relative expression level of ABA responsive gene *NbNCED*. WT: wild-type *N. benthamiana*. OE1 and OE2: different transgenic *N. benthamiana* lines overexpressed *ScDREB2B-1*. All data points were means ± standard error (*n* = 3). Significant differences were found between different letter substitutes on the column calculated by Duncan’s new multiple range test (*p*-value < 0.05).

Under normal conditions, there was no significant difference in the ABA content between the transgenic lines and the WT plants (Figure 8A). Interestingly, the ABA contents of OE1 and OE2 was higher than that of the WT plants after 2 days of drought treatment, which were 1.24 and 1.13 times that of the WT plants, respectively. The RT-qPCR results showed that there was no significant difference in the expression level of the ABA response gene *NbNCED* between the WT plants and transgenic lines under normal conditions (Figure 8B). However, after 2 days of drought treatment, the expression level of *NbNCED* in the two transgenic lines was significantly higher (1.99 and 4.20 times) than that of the WT plants (Figure 8B). The above results showed that ABA accumulated, and the expression of its response gene (*NbNCED*) increased in transgenic plants under drought stress, indicating that *ScDREB2B-1* could aid in improving the tolerance of *N. benthamiana* plants to drought stress by regulating the ABA signal.

### 2.7. Overexpression of the ScDREB2B-1 Upregulated Reactive Oxygen Species Scavenging Enzyme Activity and the Expression Level of Stress-Responsive Genes in N. benthamiana under Drought Conditions

**Figure 9:**
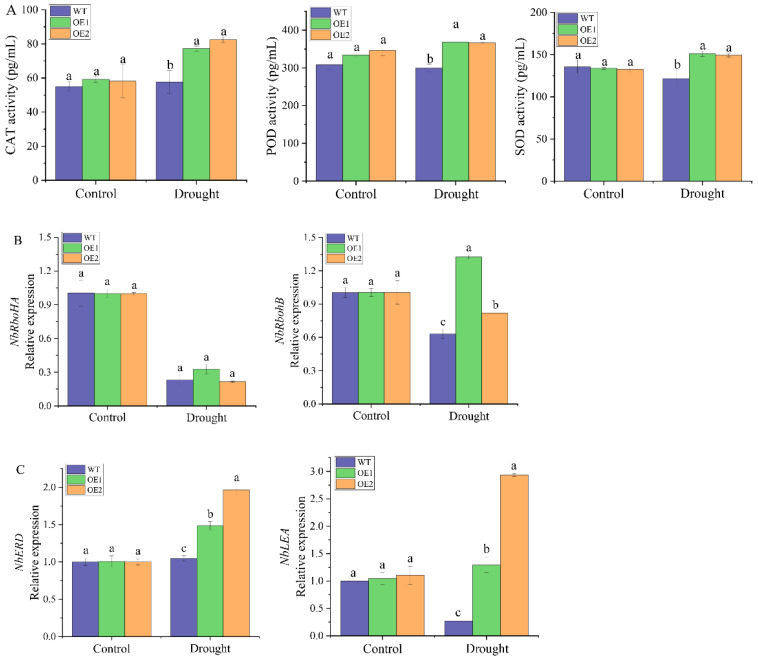
Determination of physiological index and expression level of stress-related genes in transgenic *Nicotiana benthamiana* plants overexpressed the *ScDREB2B-1* gene under drought stress for 2 d. (**A**) Activities of catalase (CAT), peroxidase (POD) and superoxide dismutase (SOD). (**B**) Relative expression level of reactive oxygen species (ROS) synthase-related genes *NbRbohA* and *NbRbohB*. (**C**) Relative expression level of osmotic stress-related genes *NbERD* and *NbLEA*. WT: wild-type *N. benthamiana*. OE1 and OE2: different transgenic *N. benthamiana* lines overexpressed *ScDREB2B-1*. All data points were means ± standard error (*n* = 3). Significant differences were found between different letter substitutes on the column calculated by Duncan’s new multiple range test (*p*-value < 0.05).

Under normal conditions, there was no difference in catalase (CAT), peroxidase (POD) and superoxide dismutase (SOD) activities between transgenic lines and the WT plants (Figure 9A). After 2 days of drought treatment, the CAT, POD and SOD activities of all transgenic plants were significantly increased, which were 1.34 and 1.43 times, 1.23 and 1.22 times and 1.24 and 1.23 times of the WT plants, respectively. The expression levels of four stress-responsive genes in the WT plants and transgenic lines under drought stress were analyzed by RT-qPCR (Figure 9B,C). The results showed that there was no significant difference in the expression levels of these four genes between the WT plants and transgenic lines under normal conditions. Two days after drought stress, the expression level of the reactive oxygen species (ROS)-producing enzyme-related gene *NbRbohA* in the transgenic lines was not significantly different from that in the WT plants (Figure 9B). In contrast, expression of the *NbRbohB* gene in the OE1 and OE2 transgenic lines was 2.10 and 1.30 times higher than that in the WT plants, respectively (Figure 9B). Compared with the WT plants, the osmotic stress-related genes *NbERD* and *NbLEA* were significantly upregulated in the two transgenic lines, which were 1.05 and 1.88 times and 4.78 and 10.86 times that of the WT plants, respectively (Figure 9C). In general, under drought stress, the overexpression of *ScDREB2B-1* significantly upregulated the expression of ROS production enzyme-related genes (*NbRbohB*) and osmotic stress-related genes (*NbERD* and *NbLEA*), as well as significantly increased ROS-scavenging enzyme activity (CAT, POD and SOD).

### 2.8. Proposed Model for the Overexpression of Sugarcane ScDREB2B-1-Mediated Regulation of N. benthamiana Plants under Drought Stress

**Figure 10:**
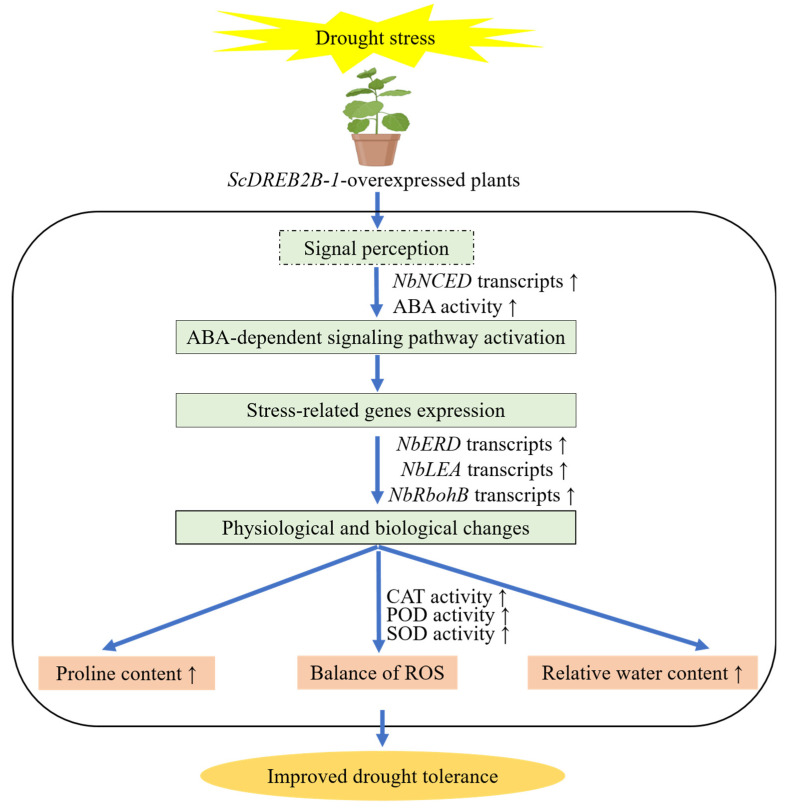
Proposed working model for the sugarcane *ScDREB2B-1* overexpression-mediated regulation of *Nicotiana benthamiana* under drought stress. *NbNCED*: nine-cis-epoxycarotenoid dioxygenase gene; ABA: abscisic acid; *NbERD*: early responsive to dehydration gene; *NbLEA*: late-embryogenesis-abundant protein; *NbRbohB*: respiratory burst oxidase homolog protein B; CAT: catalase; POD: peroxidase; SOD: superoxide dismutase; ROS: reactive oxygen species. The up arrow indicated that the detected physiological and biochemical indexes and gene expression level in the transgenic plants were higher than that in the wild-type plants under drought stress.

Based on these results, the sugarcane *ScDREB2B-1* gene could enhance the drought resistance of *N. benthamiana* plants by regulating the expression of stress response genes, together with physiological and biochemical reactions. Collectively, compared with WT plants, the overexpression of the *ScDREB2B-1* gene improved the drought tolerance of transgenic *N. benthamiana* by activating the ABA signaling pathway to enhance the expression of the ABA response gene (*NbNCED*) and the ABA content, upregulating the expression level of the stress-related genes (*NbRbohB*, *NbERD* and *NbLEA*) and then changing the physiological and biochemical levels of the plants, such as modulating the intracellular ROS level through the enhanced activities of CAT, POD and SOD and increasing the relative water and proline content (Figure 10).

## 3. Discussion

DREB is a multigene family that can be classified into six groups (A-1–A-6) [7,8]. As reported, the functions of different genes in the DREB (A-2) subgroup are different. For example, transgenic tobacco plants with the *Pennisetum glaucum PgDREB2A* gene have a tolerance to high ion and high osmotic stress [38]. Overexpressing the *G. hirsutum* *GhDREB1* gene in tobacco enhanced the tolerance of transgenic plants to low temperature stress [39]. Matsukura et al. [29] found that the expression of the *O. sativa OsDREB2A* gene was upregulated under high temperature, drought and high salt stress but not induced by low temperature stress. In contrast, the *OsDREB2B* gene, but not *OsDREB2C*, was strongly induced only under low temperature stress [29]. The overexpression of the *A. mongolicus* *AmDREB2C* gene in *A. thaliana* enhanced the low temperature tolerance, high temperature tolerance and drought resistance by regulating the fatty acid composition and inducing the expression of stress-induced genes [40]. Therefore, identifying sugarcane *DREB* gene family members and revealing their biological functions are of great significance in sugarcane stress-resistance breeding.

The expression level of *DREB* genes can be induced by adversity stress in plants. Under drought conditions, the *SbDREB2* gene could be rapidly induced by drought treatment, and its gene expression abundance in the salt-tolerant sugarcane variety was significantly higher than that in the salt-sensitive sugarcane variety [41]. The expression level of the A-2 group gene *FvDREB6* in *Fragaria vesca* was increased in young leaves under drought stress [42]. Ribonucleic acid gel blot analysis demonstrated that the stress-induced *RD29A* promoter could effectively induce *Z. mays ZmDREB2A* expression under low temperature and drought stress and that *ZmDREB2A* was further validated to play an important role in the drought tolerance of plants [43]. In the present study, ScDREB2B-1 and ZmDREB2A were clustered in the same evolutionary branch (Figure 2), likely reflecting their similar functions. The transcripts of *ScDREB2B-1* were induced by PEG, NaCl and ABA, and the gene expression levels were significantly upregulated to varying degrees in different time periods (Figure 3), indicating that the *ScDREB2B-1* gene in sugarcane may rely on the ABA signal transduction pathway to respond to drought and salt stress.

Plants can reduce or eliminate the damage under drought stress and enhance their drought resistance by improving plant growth, RWC, water loss rate and other morphological, physiological and gene expression adaptations [40,44,45,46,47]. Mallikarjuna et al. [45] showed that the two lines of *OsDREB2A* transgenic rice had significant tolerance to drought stress, while the surviving WT seedlings were short and pale green under drought stress. In this study, the RWC of *ScDREB2B-1* transgenic plants was significantly higher than that of WT plants under drought stress for 2 d (Figure 7B). As reported, RWC is expected to be used for the large-scale assessment of drought-induced mortality (DIM) risk and is an accurate predictor of drought mortality risk, which has special significance [27,48]. Consistent with our study, the RWC of the transgenic *Lotus corniculatus* with *Populus euphraria PeDREB2a* and *Kandelia candel KcERF* genes was also significantly higher than that of WT plants under drought treatment [47]. These data reveal that *ScDREB2B-1* could increase the RWC of transgenic plants, thus improving their water retention capacity and drought tolerance.

Proline is thought to be an osmotic protectant that can help crops to stabilize subcellular structure, scavenge free radicals, maintain enzyme structure and scavenge ROS under water stress [49]. After drought treatment, the control plants were severely wilted, while the *CgDREBa* transgenic *Chrysanthemum grandiflorum* lines were less affected, with a higher proline content [44]. In transgenic tobacco, overexpression of the *G. max GmDREB2* gene activated the expression of DRE-containing downstream genes *Rd29A* and *cor15a*, and the level of free proline accumulated under drought conditions [50]. Similarly, our study showed that the proline content of *ScDREB2B-1* transgenic tobacco was higher than that of the WT plants (Figure 7C). This indicates that the *ScDREB2B-1* gene could regulate proline accumulation, which may function in defense and turgor pressure maintenance against water-deprived conditions.

ABA is an important signal molecule in the plant signal transduction pathway. Research has shown that DREB (A-2) genes can play a role in abiotic stress responses through ABA-dependent and ABA-independent signal transduction pathways [17,40,46,50,51]. For example, the expression level of the *P. euphratica PeDREB2* gene can be induced by low temperature, drought, high temperature and salt stress but not by ABA hormones [51]. The transcripts of the sorghum DREB (A-2) subgroup gene *SbDREB2* can be rapidly induced not only by drought and high salt stress but also by ABA stimulus [37]. *Vigna radiata VrDREB2A* was involved in ABA-dependent signal pathways, and its expression level was induced 1 h after ABA treatment and reached a peak at 24 h [46]. Haake et al. [17] found that the expression level of CBF/DREB1 gene *CBF4* increased under drought and exogenous ABA treatments but decreased significantly in the *aba1-1* mutant lacking ABA response elements, indicating that ABA biosynthesis is necessary for *CBF4* gene expression under drought stress [17]. In this study, the promoter of *ScDREB2B-1* contained many cis-acting elements involved in the ABA- and drought-responsive pathways (Figure 4A). The expression level of *ScDREB2B-1* peaked at 6 h in the leaves and roots of ROC22 after 100 μM ABA treatment (Figure 3). In addition, compared to the WT plants, the ABA content increased in transgenic *N. benthamiana* plants overexpressing *ScDREB2B-1* (Figure 8A), and the expression level of the ABA-responsive gene *NbNCED* increased under drought stress (Figure 8B). These data reveal that the *ScDREB2B-1* gene plays a role in plant drought resistance depending on the ABA pathway. However, as *ScDREB2B-1* participates in the ABA-dependent signaling pathway, further investigation is needed.

Under abiotic stress, plant cells will have oxidative bursts and produce a large number of ROS, such as SOD, hydrogen peroxide (H_2_O_2_) and peroxide free radicals, which break the dynamic ROS balance and eventually lead to secondary oxidative stress in plant cells [52]. ROS clearance is mainly carried out through antioxidant enzyme-promoting systems and nonenzymatic systems, in which enzymatic antioxidants include APX, CAT, POD, SOD and GST [27,48,52]. The determination of antioxidant enzyme activity or the analysis of its gene expression level under drought stress can effectively evaluate the involvement of antioxidant enzyme-promoting systems under drought stress. In this study, under drought stress, the enzymatic activity of CAT, POD and SOD in transgenic *N. benthamiana* overexpressing *ScDREB2B-1* were significantly higher than those in WT plants (Figure 9A). Similarly, compared to WT plants, the drought resistance of tobacco overexpressing the *M. pumila MdDREB76* gene was enhanced with higher activities of ROS-scavenging enzymes (APX, CAT and SOD) and the increased expression level of ROS-eliminating enzyme-related genes [27]. Compared with WT plants, overexpression of *Daucus carota DcDREB1A* helped to regulate the activity of ROS scavenging enzymes (SOD and POD) to maintain ROS homeostasis in transgenic *A. thaliana* plants under drought treatment, reduce stomatal pore size and density and regulate lignin synthesis to control water loss [53]. As reported, the increased expression levels of *NbRbohA* and *NbRbohB* can lead to O^2−^ and H_2_O_2_ accumulation [54]. H_2_O_2_ was considered a key factor in cell-programmed death, which can inactivate the enzyme through its mercaptan group and can also act as a diffusible signal molecule to play a vital role in the stress resistance of plant cells [55]. In our study, the transcript level of *NbRbohB* in *ScDREB2B-1* transgenic plants was higher than that in WT plants under drought stress (Figure 9B). These results suggest that *ScDREB2B-1* may be involved in drought stress by regulating the ROS pathway.

The expression level of osmotic stress-related genes *NbERD* and *NbLEA* is related to drought resistance [27,40,46,55]. Under drought conditions, the expression level of downstream genes related to drought stress response (*AtRD17*, *AtRD29A* and *AtRD29B*) in *Arabidopsis* plants overexpressing *V. radiata VrDREB2A* increased, and the drought tolerance of transgenic plants was enhanced [46]. In this study, the expression levels of *NbERD* and *NbLEA* in transgenic plants were significantly increased under drought stress for 2 d (Figure 9C). Similar expression profiles of the upregulated *NtERD10B/NtERD10D/NtLEA* and *AtRD29A/AtCOR15A/AtLEA7* genes in transgenic tobacco overexpressing *M. domestica MdDREB76* [27] and *M. prunifolia* overexpressing *MpDREB2A* [55], respectively, have also been observed under dehydration. These results demonstrate that *ScDREB2B-1* may confer increased drought resistance by positively regulating the expression of osmotic stress-related genes *NbERD* and *NbLEA*. As reported, the copy number and location of the transgene, together with codon bias, have an impact on the gene expression level [56,57]. In stable genetically modified organisms, the expression level of transgenes is affected by many factors, especially the copy number of transgenes [58,59]. In addition, due to the complex effects of integrating multiple copies in tandem sequences, the copy number of transgenes does not always have a good correlation with the level of transgene expression [60]. Codon usage bias (CUB) exists in the genomes of almost all species, which is the result of the combined effects of mutation, selection and drift of genes and species over a long period of evolution [61]. By analyzing the codon usage preferences of the plant chloroplast genome and selecting the optimal codon, the transformation efficiency and expression level of genes can be effectively improved. A clearer understanding of the factors influencing transgene expression would improve the growth and development of plants. Thus, the correlation of *ScDREB2B-1* transgene expression with copy number, the sequence character of the insertion site or codon bias can be further examined by techniques including southern blot, absolute quantitative real-time PCR, genome walking and codon bias analysis.

## 4. Materials and Methods

### 4.1. Cloning and Sequence Analysis of the Sugarcane ScDREB2B-1 Gene

Based on our previous transcriptome database of sugarcane under cold stress (unpublished), a putative *DREB* unigene sequence (c151724.graph_c0) was mined and named *ScDREB2B-1*. The NCBI Primer Blast program (https://www.ncbi.nlm.nih.gov/tools/primer-blast/) (accessed on 1 August 2017) was used to design the cloning primers for *ScDREB2B-1* (pMD19-T-*ScDREB2B-1*-F/R, Table S1). The cDNA from the leaves of ROC22, which was reversed by a RevertAid First Strand cDNA Synthesis Kit (Thermo Fisher Scientific, Shanghai, China), was used as a template for PCR amplification (Table S2). The target fragment was then purified, ligated with the pMD19-T vector, transformed into *Escherichia coli* DH5α competent cells and sent for sequencing (Boshang Biotechnology Co., Ltd., Shanghai, China). The positive plasmid named pMD19-T-*ScDREB2B-1* was stored at −80 °C.

The physical and chemical properties of the protein encoded by the *ScDREB2B-1* gene were analyzed using online ProParam software Expasy (https://web.expasy.org/protparam/) (The SIB Swiss Institute of bioinformatics, Switzerland, accessed on 1 August 2017). A total of 51 DREB proteins (A1 to A6 subgroup members of DREB subfamily) from *A. thaliana*, *O. sativa*, *Z. mays*, *S. bicolor*, *S. spontaneum*, *L. chinensis*, *V. unguiculata* and *Hordeum vulgare* were retrieved from the NCBI Entrez database (http://www.ncbi.nlm.nih.gov) (accessed on 4 August 2021), UniProt (https://www.uniprot.org/) (accessed on 4 August 2021) and maize genome sequence database (version 5b.60, http://www.maizegdb.org/) (accessed on 4 August 2021) [8,62] (Table S3). Then, the phylogenetic tree was constructed by Neighbor-Joining (NJ) (BootStrap 1000) using MEGA 6 software (accessed on 24 December 2021) [8]. ITOL software (https://itol.embl.de/) (accessed on 24 December 2021) was used to draw the phylogenetic tree.

### 4.2. Sugarcane Materials and Stress Treatments

The sugarcane hybrid ROC22, which is the main sugarcane cultivar in mainland China, was collected from the Key Laboratory of Sugarcane Biology and Genetic Breeding, Ministry of Agriculture and Rural Affairs (Fuzhou, China). According to the method of Chen et al. [63], the stems of 10-month-old ROC22 were soaked overnight with 50% carbendazim and cultured in sand in a 30 °C incubator (light 13 h/dark 11 h; illumination: light 22,000 Lx/dark 0 Lx; humidity 80%). When the first fully expanded leaf appeared, the sand was removed for rehydration. The sugarcane seedlings were then transferred to Hoagland nutrient solution [64] and cultured on 3–4 fully expanded leaves for treatment. The control group was cultured in a normal Hoagland nutrient solution [64], and the stress groups were cultured in a Hoagland nutrient solution containing 20% PEG 8000 or 250 mM NaCl or sprayed with 100 µM ABA on sugarcane leaves. Then, +1 leaf with a visible dewlap (the collar between the leaf blade and sheath) and roots were collected at 0, 0.5, 3 and 6 h. Each biological replicate was a mixture of three plants. Three biological replicates were set up in the experiment. The samples were frozen in liquid nitrogen and stored at −80 °C for use.

### 4.3. Expression Analysis of ScDREB2B-1 in Sugarcane under Different Stressors

The total RNA of the samples was extracted using the Trizol method. The DNA in the RNA sample was removed according to the instructions of the RQ1 RNase-Free DNase kit (Promeaga, Madison, WI, USA). The first-strand cDNA was synthesized by reverse transcription using the Prime-Script RT-PCR Kit (TaKaRa, Dalian, China) and used as a template for RT-qPCR detection (Table S2). Based on the *ScDREB2B-1* gene sequence, its specific quantitative primers RT-qPCR-*ScDREB2B-1*-F/R (Table S1) were designed using the NCBI online website Primer Blast (https://www.ncbi.nlm.nih.gov/tools/primer-blast/) (accessed on 1 August 2017). *CAC* (clathrin adaptor complex) and *CUL* (cullin) genes (Table S1) were selected as internal reference genes [65] to analyze the expression pattern of the *ScDREB2B-1* gene in leaves and roots of ROC22 treated with 20% PEG 8000, 250 mM NaCl and 100 μM ABA. RT-qPCR analysis was performed in an ABI 7500 Fast Real-time PCR amplification system (Applied Biosystems, Waltham, MA, USA), and three technical replicates for each sample were valuated. The 2^−^^△△CT^ algorithm was applied to analyze the relative gene expression level, and DPS 9.50 was used to calculate significant differences in the data (*p*-value < 0.05).

### 4.4. Promoter Cloning of the ScDREB2B-1 Gene and Expression Profile Analysis of Its Homologous Gene in S. spontaneum under Drought Treatment

A homologous gene (*SsAP2/ERF-107*) of *ScDREB2B-1* in the *S. spontaneum* SsAP2/ERF gene family [63] was selected by DNAMAN 6.0 analysis. The upstream 3000 bp promoter sequence of *SsAP2/ERF-107* was used to design the primers of Promoter-*ScDREB2B-1*-F/R (Table S1) using Primer premier 5.0 software (Primer premier, San Francisco Bay Area, CA, USA). The genomic DNA of ROC22 leaves was applied to the promoter cloning of the *ScDREB2B-1* gene using the homologous cloning method (Table S2). Promoter elements of *ScDREB2B-1* were predicted and analyzed by PlantCARE (http://bioinformatics.psb.ugent.be/webtools/plantcare/html/) (accessed on 24 October 2019). The original RNA sequencing data (PRJNA590595) of *S. officinarum* (Co 06022) after 0, 2, 6 and 10 d and 10 d of drought stress recovery were downloaded from the sequence reading archive database (https://www.ncbi.nlm.nih.gov/sra/) (accessed on 1 May 2021). Fastp [66] and hisat2 [67] programs were used to improve sequence quality and map sequence data to the genome of *S. spontaneum* [68]. Log_2_ (FPKM + 1) of fragments per kb per million (FPKM) was used to represent the gene expression of *S. spontaneum SsERF* genes under drought stress, and TBtools software (accessed on 21 December 2021) [69] was applied to visualize the data.

### 4.5. Transcriptional Self-Activation Activity Assay

The Matchmaker Gold Yeast Two-Hybrid System was used to detect the transcriptional self-activation activity of the ScDREB2B-1 protein. The primers pGBKT7-*ScDREB2B-1*-F/R with *Eco*RI and *Bam*H I restriction sites (Table S1) were designed to amplify the complete coding domain sequence (267 aa). These target fragments were purified and ligated to the pGBKT7 vector with the In-Fusion HD cloning enzyme (TaKaRa, Dalian, China). After sequencing, the plasmids, pGBKT7-*ScDREB2B-1*, pGBKT7-53+pGADT7-T (positive control) and empty pGBKT7 (negative control) were transferred to yeast cells Y2H Gold and spread on SD-Trp (SDO) solid medium (positive control was spread on SD/-Trp/-Leu (DDO) solid medium) at 30 °C for 2–3 d. The cell solution, cultured in SDO liquid media at 30 °C for 24 h, was diluted 10-, 100- and 1000-times. Then, 9 μL diluent was cultured in SD/-Trp (SDO), SD/-Trp/X-α-Gal (SDO/X) and SD/-Trp/X-α-Gal/AbA (SDO/X/A) solid media at 30 °C for 96 h and observed.

### 4.6. Production of Transgenic Seedlings

The primers CD3689-*ScDREB2B-1*-F/R (Table S1) were designed according to the sequences of the *ScDREB2B-1* gene and the Gateway entry vector pDNOR-221. The pMD19-T-*ScDREB2B-1* plasmid was used as a template for PCR amplification (Table S2). After gel recovery and purification, the target fragment was used to construct the intermediate vector pDNOR-221-*ScDREB2B-1* based on the Gateway BP reaction method (Invitrogen, Carlsbad, CA, USA). The recombinant plant overexpression vector CD3689-*ScDREB2B-1* was then obtained using the Gateway LR reaction (Invitrogen, USA). Due to the low genetic transformation efficiency and long growth period of sugarcane [70], the functional analysis of sugarcane genes can be carried out using transformed model plants. Therefore, the *ScDREB2B-1* gene was genetically transformed into *N. benthamiana* by *Agrobacterium tumefaciens*-mediated leaf disc infection [71]. After selecting the transformants on a selective Murashige and Skoog (MS) medium containing 100 μL/L herbicide (Sangon Biotech, Shanghai, China), soil cultivation and PCR identification (Table S2), nine homozygous transgenic *N. benthamiana* seedlings from the T_3_ generation were obtained.

### 4.7. Phenotypic Analysis of Transgenic N. benthamiana under Drought Stress

To functionally characterize *ScDREB2B-1*, two homozygous transgenic lines, OE1 and OE2, were randomly selected from T_3_ generation seeds that overexpressed the *ScDREB2B-1* gene. The transgenic lines and the wild-type *N. benthamiana* (WT) plants were grown on MS medium for 14 days. The seedlings were then cultured in aseptic water for 3 days and transplanted into flowerpots (nutrient soil: vermiculite = 3:1) for 2 weeks with quantitative watering management at 23 °C (light 13 h/dark 11 h; illumination: 22,000 Lx/dark 0 Lx; humidity 80%). At the five-leaf stage, the plants withheld water for drought stress. The plant growth status was photographed before treatment (0 d) and 2 days after drought treatment with phenotype emergence. The target fragment of the *ScDREB2B-1* gene in transgenic *N. benthamiana* plants under drought treatment for 0 and 2 days was detected by RT-PCR analysis (Figure S2A,B). Tobacco leaves were collected for the determination of chlorophyll content, relative water content (RWC), ABA content, physiological indexes related to stress resistance and the expression level of stress response genes.

### 4.8. Measurements of the Physiological and Biochemical Indexes in Transgenic Plants

Under drought stress for 0 and 2 d, the contents of chlorophyll, RWC, ABA and proline, and the activities of CAT, POD and SOD in the WT and transgenic plants, were measured. The chlorophyll content in leaves of the WT and transgenic plants were detected three times in each plant by a hand-held chlorophyll meter (Minolta Camera Co., Ltd., Tokyo, Japan). For the RWC measurement, the fresh weight (FW) of the leaf was recorded. Then, the leaf was immersed in distilled water for 4 h and weighed as the turgid weight (TW). After drying for 48 h at 80 °C, the dry weight (DW) of the leaf was recorded. The RWC of the leaf was calculated by a formula of RWC% = (FW − DW)/(TW − DW) × 100 [72]. The contents of ABA and proline, and the activities of CAT, POD and SOD in leaves of the WT and transgenic plants, were determined, respectively, by the enzyme-linked immunosorbent assay kit (Jiangsu Meibiao Biotechnology Co., Ltd., Yancheng, China).

### 4.9. Expression Analysis of Stress Response Genes in Transgenic Plants

The leaf total RNA of the WT and transgenic plants under drought stress for 0 and 2 d was extracted using the Trizol method and reversed to the first-strand cDNA by using Hifair^®^ II 1st Strand cDNA Synthesis Kit (gDNA digester plus) (Yeasen Biotechnology Co., Ltd., Shanghai, China). The expression level of the *ScDREBB-1* gene and the stress response genes was analyzed using the RT-qPCR method (Table S2). There was no significant difference in the transcript level of the *ScDREB2B-1* gene between OE1 and OE2, suggesting similar expression levels of *ScDREB2B-1* in transgenic lines (Figure S2C). The stress response genes, including ABA response genes (ABA response binding element protein (*NbAREB*) and nine-cis-epoxycarotenoid dioxygenase gene (*NbNCED*)), ROS-generated enzyme-related genes (respiratory burst oxidase homolog protein A (*NbRbohA*) and B (*NbRbohB*)), osmotic stress-related genes (early responsive to dehydration (*NbERD*) and late-embryogenesis-abundant protein (*NbLEA*)) [54] (Table S1). *NbEF-1α* was used as the internal reference gene to normalize the quantitative data. RT-qPCR analysis was performed on an ABI 7500 Fast Real-time PCR amplification system (Applied Biosystems, USA), and three technical replicates for each sample were set. The 2^−^^△△CT^ algorithm was applied to analyze the relative gene expression level, and the DPS 9.50 was used to calculate the significant difference in the data (*p*-value < 0.05).

## 5. Conclusions

In this study, a new sugarcane *ScDREB2B-1* gene belonging to the DREB (A-2) subgroup and having transcriptional self-activation activity was characterized. The expression level of *ScDREB2B-1* was upregulated in the roots and leaves of sugarcane ROC22 after exposure to PEG, NaCl and ABA. In addition, the overexpression of *ScDREB2B-1* conferred drought tolerance to transgenic *N. benthamiana* plants and affected several physiological and biochemical indices and the expression of stress-response genes. *ScDREB2B-1* may mediate drought responses by activating the ABA pathway to enhance ABA content and the transcript level of ABA response genes, modulate antioxidant enzyme activities, alter ROS-related gene expression and elevate the relative water content, proline content and the expression level of osmotic stress-related genes. These findings enhance the understanding of the role of sugarcane *ScDREB2B-1* in abiotic stress response and provide a theoretical basis for stress resistance breeding in sugarcane.

## Data Availability

Not applicable.

## Supplementary Materials

The supporting information can be downloaded at: https://www.mdpi.com/article/10.3390/ijms23179557/s1.
