# Supplementary material for: Sugarcane ScDREB2B-1 Confers Drought Stress Tolerance in Transgenic Nicotiana benthamiana by Regulating the ABA Signal, ROS Level and Stress-Related Gene Expression
Source: Int J Mol Sci. 2022 Aug 23;23(17):9557. doi: 10.3390/ijms23179557 (PMC9455921; doi:10.3390/ijms23179557)
Supplement: Supplementary file 1 [file ijms-23-09557-s001.zip › ijms-1850382-supplementary.pdf]

Sugarcane *ScDREB2B-1* Confers Drought Stress Tolerance in Transgenic *Nicotiana benthamiana* by Regulating the ABA Signal, ROS Level and Stress-related Gene Expression

Yufeng Chen[1], Zhu Li[1], Tingting Sun[1], Dongjiao Wang[1], Zhoutao Wang[1], Chang Zhang[1], Youxiong Que[1], Jinlong Guo[1], Liping Xu[1,*] and Yachun Su[1,2,*]

1 Key Laboratory of Sugarcane Biology and Genetic Breeding, Ministry of Agriculture and Rural Affairs, College of Agriculture, Fujian Agriculture and Forestry University, Fuzhou, Fujian, 350002, China; cyf9410@163.com (Y.C.); lizhu7799@163.com (Z.L.); sunting3221@163.com (T.S.); dongjiaow@126.com (D.W.); wzt1417@126.com (Z.W.); zchang1997@163.com (C.Z.); queyouxiong@126.com (Y.Q.); jl.guo@163.com (J.G.)

2 Key Laboratory of Sugarcane Biotechnology and Genetic Improvement (Guangxi), Ministry of Agriculture and Rural Affairs/Guangxi Key Laboratory of Sugarcane Genetic Improvement/Sugarcane Re-search Institute, Guangxi Academy of Agricultural Sciences, Nanning 530007, China

* Correspondences: xlpmail@126.com (L.X.); syc2009mail@163.com (Y.S.); Tel.: +86-591-8385-2547 (L.X. & Y.S.)

Supplementary Figures and Tables

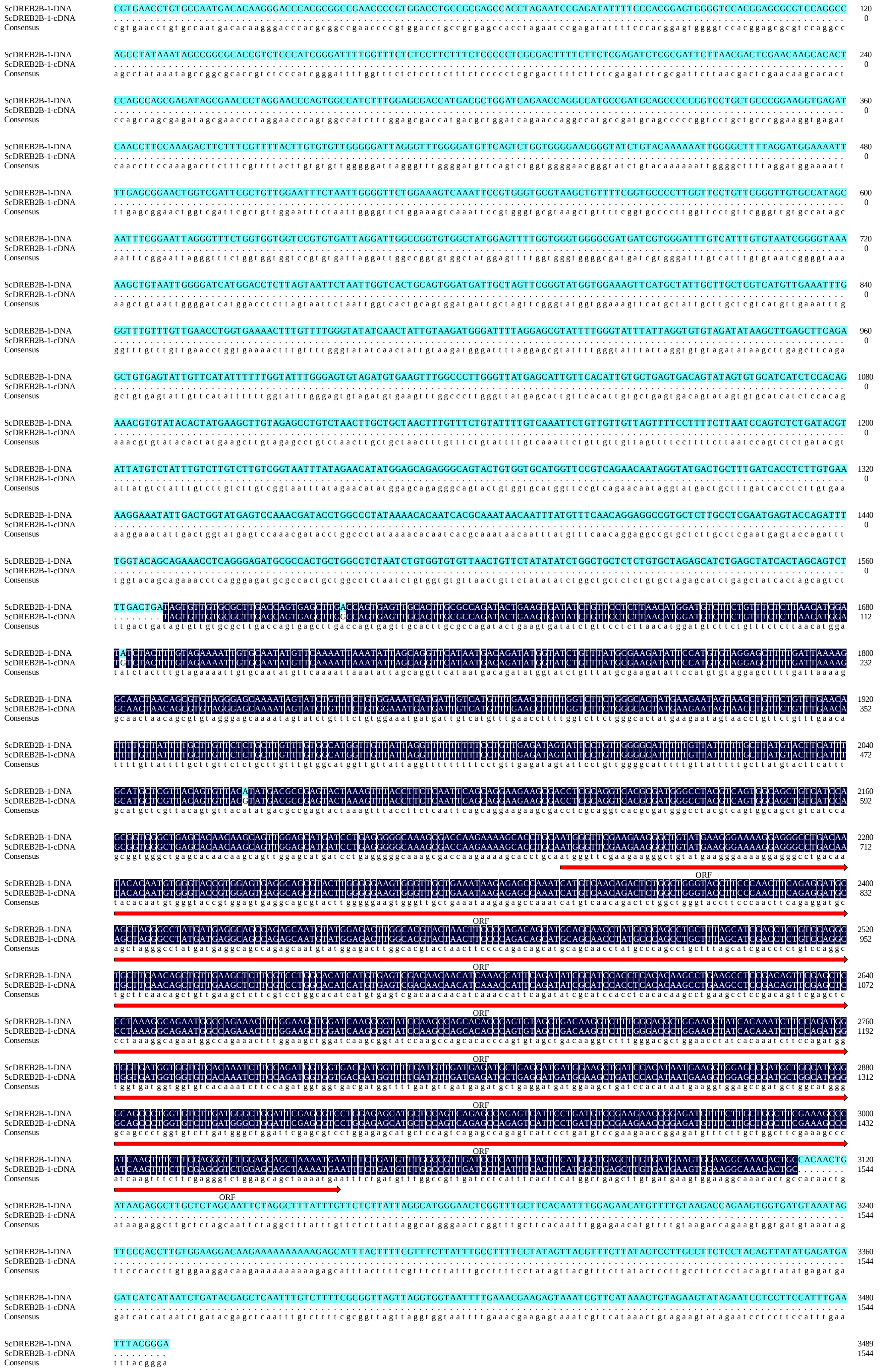

Figure S1. Nucleic acid sequence alignment of the cDNA and DNA sequences of *ScDREB2B-1* gene. The red arrow was the ORF sequence.

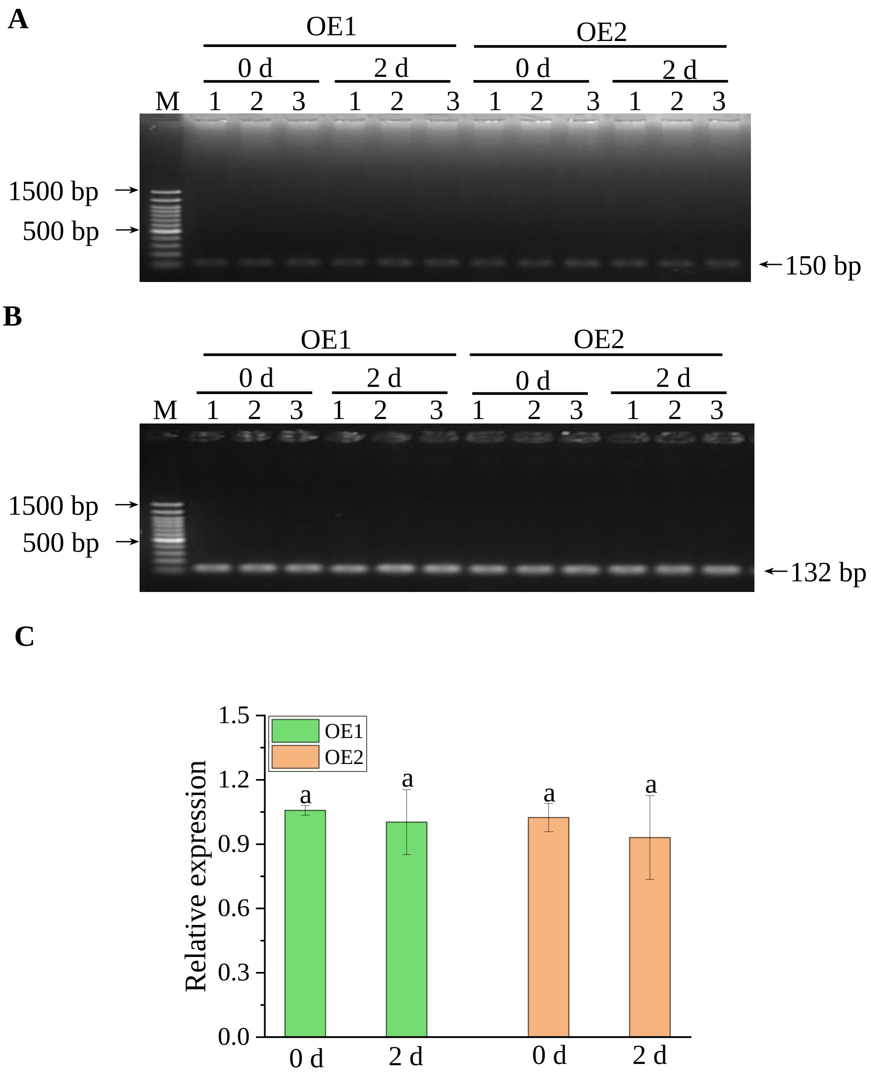

Figure S2 Detection of the *ScDREB2B-1* gene in two transgenic *Nicotiana benthamiana* lines under drought stress for 2 d. (A) RT-PCR analysis of the *NbEF-1α* gene with the primers *NbEF1α*-F/R (Table S1); (B) RT-PCR analysis of the *ScDREB2B-1* gene with the primers RT-qPCR-*ScDREB2B-1*-F/R (Table S1). M: 100 bp-Ⅲ DNA marker; OE1 and OE2: different transgenic *N. benthamiana* lines overexpressed *ScDREB2B-1*. 1-3: PCR amplification products of different duplicates transgenic *N. benthamiana* plants overexpressed *ScDREB2B-1*. (C) The relative expression level analysis of the *ScDREB2B-1* gene by RT-qPCR method. *NbEF-1α* was used as the internal reference gene. All data points were means ± standard error (n=3). Significant differences were found between different letter substitutes on the column calculated by Duncan's new multiple range test (P-value < 0.05).

Table S1 Primers used in this study

Primer name	Primer sequences (5'-3')
pMD19-T-*ScDREB2B-1*-F	TAGTGTTGTGCGCTTGACCA
pMD19-T-*ScDREB2B-1*-R	GCAGTGTTTGCCTTCCACTT
RT-qPCR-*ScDREB2B-1*-F	ACACAATGTGGGTACCGTGG
RT-qPCR-*ScDREB2B-1*-R	ATAGGCCCTAGCTGCATCCT
Promoter-*ScDREB2B-1*-F	CGTGAACCTGTGCCAATG
Promoter-*ScDREB2B-1*-R	CTCCCGTAAATTCAAATGG
pGBKT7-*ScDREB2B-1*-F	CATGGAGGCCGAATTCATGGGTTCGAAGAAGGGCTG
pGBKT7-*ScDREB2B-1*-R	GGATCCCCGGGAATTCTTTTAGCTGCTCCAGACCCT
CD3689-*ScDREB2B-1*-F	GGGGACAAGTTTGTACAAAAAAGCAGGCTTCATGGGTTCGAAGAAGGGCTG
CD3689-*ScDREB2B-1*-R	GGGGACCACTTTGTACAAGAAAGCTGGGTCTTTTAGCTGCTCCAGACCCT
NbEF1α-F	TGCTGCTGTAACAAGATGGATGC
NbEF1α-R	GAGATGGGGACAAAGGGGATT
NbRbohA-F	ACACACGCCATCAGAACTCCA
NbRbohA-R	CCCACCCAACCAAAATACGC
NbRbohB-F	GTTTGCCAGCCACCACCTAAT
NbRbohB-R	AAGAGCAGAACGAGCATCACC
NbERD-F	CACTGATAAGAACTATGCGTTCAC
NbERD-R	CTAAGCTAATCACATTCAGCGAG
NbLEA-F	GGATCTAATTGACAAGGCGAAG
NbLEA-R	CTCGCCGCTATAAGAGAGAG
NbNCED-F	CGACCCACGAGTCCAGATTTC
NbNCED-R	GAGCCTAGCAATTCCCGAGTG

Table S2 PCR amplification systems and programs used in this study

Test name	The PCR system	The PCR program
Cloning of *ScDREB2B-1* gene	10 × Ex Taq Buffer, 2.0 μL;<br>dNTP mixture (2.5 mM), 1.6 μL;<br>Primer-F/R (10 μM), each 1.0 μL;<br>Ex Taq enzyme (5 U/μL), 0.1 μL;<br>cDNA template, 1.0 μL;<br>ddH_2O, 13.7 μL.	94 ℃, 4 min;<br>94 ℃, 30 s, 65 ℃-55 ℃, 30 s, 72 ℃, 2 min, 10 cycles (each cycle lowers 1 ℃);<br>94 ℃, 30 s , 53 ℃, 30 s, 72 ℃, 2 min, 25 cycles;<br>72 ℃, 10 min.
RT-qPCR analysis	SYBR Green PCR Master (ROX), 10.0 μL;<br>ddH_2O, 7.0 μL;<br>Primer-F/R (10 μM), each 1.0 μL;<br>cDNA template (20-fold dilution), 1.0 μL.	50 ℃, 2 min;<br>95 ℃, 10 min; 95 ℃, 15 s, 60 ℃, 1 min, 40 cycles;<br>95 ℃, 15 min;<br>60 ℃, 1 min;<br>95 ℃, 15 s, 60 °C, 15 s.
Promoter cloning	10 × Ex Taq Buffer, 2.5 μL;<br>dNTP mixture (2.5 mM), 2.0 μL;<br>Primer-F/R (10 μM), each 1.0 μL;<br>Ex Taq enzyme (5 U/μL), 0.125 μL;<br>DNA template, 1.0 μL;<br>ddH_2O, 17.375 μL.	94 ℃, 4 min;<br>94 ℃, 30 s, 55 ℃, 30 s, 72 ℃, 3 min 30 s, 35 cycles;<br>72 ℃, 10 min.
Construction of *ScDREB2B-1* entry vector	10 × Ex Taq Buffer, 2.5 μL;<br>dNTP mixture (2.5 mM), 2.0 μL;<br>Primer-F/R (10 μM), each 1.0 μL;<br>Ex Taq enzyme (5 U/μL), 0.125 μL;<br>cDNA template (pM19-T-*ScDREB2B-1* plasmid), 1.0 μL;<br>ddH_2O, 17.375 μL.	94 ℃, 4 min;<br>94 ℃, 30 s, 71 ℃, 30 s, 72 ℃, 1 min, 35 cycles;<br>72 ℃, 10 min.
Target gene detection in transgentic plants	ddH_2O, 9.5 μL;<br>2 × Buffer, 12.5 μL;<br>Primer-F/R (10 μM), each 1.0 μL;<br>DNA template, 1.0 μL.	94 ℃, 4 min;<br>94 ℃, 30 s, 60 ℃, 30 s, 72 ℃, 1 min 30 s, 35 cycles;<br>72 ℃, 10 min.

Table S3 The amino acid sequences of 51 DREB proteins from A1 to A6 subgroups of DREB subfamily used for phylogenetic tree analysis

Subgroups	Protein name	Amino acid sequence
A1	AtCBF3	MNSFSAFSEMFGSDYESSVSSGGDYIPTLASSCPKKPAGRKKFRETRHPIYRGVRRRNSGKWVCEVREPNKKTRIWLGTFQTAEMAARAHDVAALALRGRSACLNFADSAWRLRIPESTCAKDIQKAAAEAALAFQDEMCDATTDHGFDMEETLVEAIYTAEQSENAFYMHDEAMFEMPSLLANMAEGMLLPLPSVQWNHNHEVDGDDDDVSLWSY
A1	HvCBF3	MDMGLEVSSSSPSSSSVSSSPEHAARRASPAKRPAGRTKFRETRHPVYRGVRRRGNTERWVCEVRVPGKRGARLWLGTYATAEVAARANDAAMLALGGRSATCLNFADSAWLLAVPSALSDLADVRRAAVEAVADFQRREAADGSLAIAVPKEASSGAPSLSPSSGSDSAGSTGTSEPSANGVFEGPVVMDSEMFRLDLFPEMDLGSYYMSLAEALLMDPPPTATIIHAYEDNGDGGADVRLWSYSVDM
A1	OsDREB1A (LOC_Os09g35030.1)	MCGIKQEMSGESSGSPCSSASAERQHQTVWTAPPKRPAGRTKFRETRHPVFRGVRRRGNAGRWVCEVRVPGRRGCRLWLGTFDTAEGAARAHDAAMLAINAGGGGGGGACCLNFADSAWLLAVPRSYRTLADVRHAVAEAVEDFFRRRLADDALSATSSSSTTPSTPRTDDDEESAATDGDESSSPASDLAFELDVLSDMGWDLYYASLAQGMLMEPPSAALGDDGDAILADVPLWSY
A1	OsDREB1B	MEVEEAAYRTVWSEPPKRPAGRTKFRETRHPVYRGVRRRGGRPGAAGRWVCEVRVPGARGSRLWLGTFATAEAAARAHDAAALALRGRAACLNFADSAWRMPPVPASAALAGARGVRDAVAVAVEAFQRQSAAPSSPAETFANDGDEEEDNKDVLPVAAAEVFDAGAFELDDGFRFGGMDAGSYYASLAQGLLVEPPAAGAWWEDGELAGSDMPLWSY
A1	ZmDREB1.2 (GRMZM2G069082_P01)	MDMGRHQLQLQHAASSSSTSASSSSEQDKPLCCSGPKKRPAGRTKFRETRHPVFRGVRRRGAAGRWVCEVRVPGRRGARLWLGTYLGAEAAARAHDAAMLALGRGAACLNFPDSAWLLAVPPPPALSGGLDGARRAALEAVAEFQRRRFGAAAADEATSGTSPPSSSSSATKPAPAIERVPVEASETVALDGAVFEPDWFGDMDLDLYYASLAEGLLVEPPPPPPPAAWDHGDCCDSGADVALWSY
A1	ZmDREB1.3 (GRMZM2G069146_P01)	MCPTKKGMTGEPSSPCRSASASTLPEHHQTVWTSPPKRPAGRTKFRETRHPVFRGVRRRGSAGRWVCEVRVPGRRGCRLWLGTFDTAEAAARAHDAAMLALAGAAACCLNFADSAWLLAVPASCASLAEVRHAVADAVEDFLRHQVVPEDDALAATPSSPSSEDGNTSDGGESSSDSSPPTGASPFEFDVFNDMSWDLHYASLAQGLLVEPPSAVTAFMDEGFADVPLWSY
A1	SsAP2/ERF-027	MCPIKKEMIGDSSSPCSSASSEHQTVWTSQPKRPAGRTKFRETRHPVFRGVRRRGNAGRWVCEVRVPGRRGCRLWLGTFDAAEAAARAHDAAMLAIAGAGRACLNFADSAWLLAVPASYASLAEVRHAVAEAVEDFLRREVVPDPEDDALSATSSTPPSSPSSSDDGSTSDGGESSDYSSPAATGAVSAFELDVFNDMSWDLYYASLAQGMLVEPPSAVTAFMDEGFADAARVAACYGVLVLTSRDVAVAAAPLGAPKPQLETRILHSPLALGLYLPRAEPTSEQQRTQLGSLRPGRAVTGRACLFRDRKNKYVARAYNLLLTKAATSLAVRDASDDDIGAPTRHGCDGAGGAEASSVRPPTSWARGQTLFIERGEPDAPKGKSDVLSAFPGSRTRSIPSKWPFRFGQNTSGRQQDCGSGIGTAATHSPHAVQVRELPGCRCPLCDTDTVGGFSIAAHKMLPSRAARPPGAEMVLARNTFLFIHS

Continued Table S3

Subgroups	Protein name	Amino acid sequence
A1	SsAP2/ERF-028	MCPIKKEMIGDSSSPCSSASSEHQTVWTSPPKRPAGRTKFRETRHPVFRGVRRRGNAGRWVCEVRVPGRRGCRLWLGTFDAAEAAARAHDAAMLAIAGAGRACLNFADSAWLLAVPASYASLAEVRHAVTEAVEDFLRREVVPDPEDDALSATSSTPPSSPSSSDDGSTSDGGESSDYSSPAATGAVSAFELDVFNDMSWDLYYASLAQGMLVEPPSAVTAFMDEGFADSKMCPIKKEMSAESSASASSWPSSSPSTSSEHHQTVWTSPPKRPAGRTKFRETRHPVFRGVRRRGNAGRWVCEVRVPGRRGCRLWLGTFDTAEAAARAHDAAMLAIAGAGACLNFADSAWLLAVPVSYASLADVRRAVAEAVEDFLQRRDGEAAAAGDTNARSATSSSAPSTSGNEDDAATDGEESSPATDTEDSSFQLDVFNDMSWDLYYASMAQGMLMELPSAVPAFGDDGDANVADVSLWSY*
A1	SsAP2/ERF-029	MCPIKKEMIGDSSSPCSSASSEHQTVWTSPPKRPAGRTKFRETRHPVFRGVRRRGNAGRWVCEVRVPGRRGCRLWLGTFDAAEAAARAHDAAMLAIAGAGRACLNFADSAWLLAVPASYASLAEVRHAVAEAVEDFLRREVVPDPEDDALSATSSTPPPSPSSSDDGSTSDGGESSDYSSPSATGAVSPFELDVFNDMSWDLYYASLAQGMLVEPPSAVTAFMDEGFADVPLWSY*
A2	ZmDREB2A (GRMZM2G006745_P01)	MTLDQNHAMPMQPPALQPGRKKRPRRSRDGPTSVAAVIQRWAERNKHLEYEESEEAKRPRKAPAKGSKKGCMKGKGGPDNTQCGYRGVRQRTWGKWVAEIREPNRVDRLWLGTFPTAEDAARAYDEAARAMYGDLARTNFPGQDATTSAQAALASTSAQAAPTAVEALQTGTSCESTTTSNYSDIASTSHKPEASDISSSLKAKCPAGSCGIQEGTPSVADKEVFGPLEPITNLPDGGDGFDIGEMLRMMESDPHNAGGADAGMGQPWCLDELDSSVLESMLQPQPEPEPFLMSEEPDMFLAGFESAGFVEGLERLN
A2	OsDREB2A (LOC_Os01g07120.1)	MERGEGRRGDCSVQVRKKRTRRKSDGPDSIAETIKWWKEQNQKLQEENSSRKAPAKGSKKGCMAGKGGPENSNCAYRGVRQRTWGKWVAEIREPNRGRRLWLGSFPTALEAAHAYDEAARAMYGPTARVNFADNSTDANSGCTSAPSLMMSNGPATIPSDEKDELESPPFIVANGPAVLYQPDKKDVLERVVPEVQDVKTEGSNGLKRVCQERKNMEVCESEGIVLHKEVNISYDYFNVHEVVEMIIVELSADQKTEVHEEYQEGDDGFSLFSY
A2	LcDREB3a	MTVHQMEAAAAAALPYAPFEIPALQPGRKKRPRRSRDGPNSVSETIRRWKEVNQQLEHDPQGAKRARKPPAKGSKKGCMQGKGGPENTQCGFRGVRQRTWGKWVAEIREPNRVSRLWLGTFPTAEVAAQAYDEAARAMYGPLARTNFPLQDAQAPAVAVPAAIEGVVRGASASCESTTTSNHSDVASSSHNKQPQAQAPEISSQSDVLESTQSVVLESTQSVVLESVRHYGQQETVPDAGSSIARSTYEEDVFEPLEPISSLPDGEADGFDIEELLRLMEADPVEVEPTTGGCWNEFQDAGANTGGSWNANTGVEMGQQEPLYLDGLDQGMLEGMLQSDYPYPMWISEDRAMHNPAFHDAEMSEFFEGL
A2	VuDREB2A	MGAYDQVSVRPLDSSRKRKSRSRGDGSRSVAETIAKWKEYNEHLYSGKDDGRSTRKAPAKGSKKGCMKGKGGPQNYECNYRGVRQRTWGKWVGEIREPNRGSRLWLGTFSTAQEAALAYDEAARAMYGPCARLNFPNISDYASVKESFKDSFLAASSCSSTTSDTTTTTTSNRSEVCAAEDAKENVLPALDKGNHSDCHKAYEYASPTSRMKLEPKDDEAGEGIIHHAEQAENVNEDQMDFSWIDALDFNDDYSKSFSNDELFQVDELLGLIENNPVDDSGLMQGLDFGQTGFPGESNPQVDDTSSSFFYQLQNPDAKLLGSLPHMEQTPSGFDYGLDFMKTVVPEDYDGGGEEPQFLNLDDVLNHDSKEMVASKE

Continued Table S3

Subgroups	Protein name	Amino acid sequence
A2	OsDREB2B (LOC_Os05g27930.1)	MTVDQRTTAKAIMPPVEMPPVQPGRKKRPRRSRDGPTSVAETIKRWAELNNQQELDPQGPKKARKAPAKGSKKGCMKGKGGPENTRCDFRGVRQRTWGKWVAEIREPNQQSRLWLGTFPTAEAAACAYDEAARAMYGPMARTNFGQHHAPAASVQVALAAVKCALPGGGLTASKSRTSTQGASADVQDVLTGGLSACESTTTTINNQSDVVSTLHKPEEVSEISSPLRAPPAVLEDGSNEDKAESVTYDENIVSQQRAPPEAEASNGRGEEVFEPLEPIASLPEDQGDYCFDIDEMLRMMEADPTNEGLWKGDKDGSDAILELGQDEPFYYEGVDPGMLDNLLRSDEPAWLLADPAMFISGGFEDDSQFFEGL
A2	ZmDREB2.2 (AC209257.4_FGP006)	MGGPDNTRHNYRGVRQRRWGKWVAEIREPNRGKRHWLGTFDNPVDAAVAYDRAAVSIHGAHAHLNFPCSAATTADASLEHEAKPMVAAAALGGGGAEAVSQRQEDDDPHDIAMYIDFDAVFDMVPFCHGIKREDCQHEGFDGDAVHSPLWALGD
A2	SsAP2/ERF-006	MDHRLPPVAMQVAAMQRQRQQQQQQFVHHLQVHQQQGTHHQPLPPAPPPPQQQQHQNSGGVGSSRAAGGRRCCPLRQSRKGCMKGKGGPDNQQCPYRGVRQRTWGKWVAEIREPNRGTRLWLGTFGSAFEAACAYDNAARKLYGDCAHLNLQLPPPAVAAMAAGGGGPAVVVSSPSPDTVAAGPAAAAGGHNRHHQYLQQQQQQAAMAAAPMMMMQYSSSYSADASSSNSGSFSNSYSSSSPVTTAAVAASPTYNYNHHQMFQMTPPPSSCSGVMMAPAVPQAQGCHVNTTTTTTTMEMQRHQQMIRELAAAPLHQEPDDFADFMTWLPEAEDFGLQGFQEVPPEVFDEAAGGIWDHTAATWSTPTMMIDSAAGAAQHQQ
A2	SsAP2/ERF-020	MVSHMDMLIRSRSNKQIDRTRQTGGGLIRNRMLCTSYVEEEHVKSTLVRAKNAVRWIFTPRIHAALITMTTNQRARLSLFGLYVLTLTGLEKARHHRTYAEHAFPAVTTFKPTPCLADHPRRRPARAHSTKPPAPLVTLSSPTPPTPIPIPMESYGRKRAWKKGPTRGKGGPQNAACEYRGVRQRTWGKWVAEIREPNKRTRLWLGSFATAEEAALAYDEAARRLYGPDAFLNLPHLRASVSAAAHQRLRWLPASARGAAAAAVPAYGLLNLNAQHNVHVIHQRLQELKNGGSPAKPPPPQPARQVAPVHHLPAAASTSPCSTVTTHAVAALPPPMSCFHALEQAVAAAAMTTADDDAEPCEGGACPPGADKPQLDLREFLQQIGVLKTDDEGMTATAKASYHGDAADAGCFGGNGEFDWDALAADLNDIAGAHGGAIGVNGGFQMDDLHEVDQFGTCLPIPVWDV*
A2	SsAP2/ERF-051	MRRKSTGPDSIAETIKWWKEQNQKLQDESGSRKAPAKGSKKGCMTGKGGPENVNCVYRGVRQRTWGKWVAEIREPNRGRRLWLGSFPTAVEAAHAYDEAAKAMYGPKARVNFSENSADANSGCTSALSLLASSVPAATLQRSDEKVETEVESVETEVHEVKTEGNDDLGSVHVACKTVDVIQSEKSVLHKEGEVSYDYFNVEEVVEMIIIELNADKKIEAHEEYHDGDDGFSLFAY
A2	SsAP2/ERF-107	MTLDQNQAMPMQPPALLPGRKKRPRRSRDGPTSVAAVIQRWAEHNKQLEHDPEGAKRPRKAPAMGSKKGCMKGKGGPDNTQCGYRGVRQRTWGKWVAEIREPNRVNRLWLGTFPTSEDAARAYDEAARAMYGDLARTNFPRQHAATYAQAALASTSVQAAPTAVEALRPGTSCESTTTSNHSDIASTSHKPEASDSSSSLKAEWPEALEAGSSGIQAGTPSVADKVFGTLEPITNLPDGGDGGVTNLPDGGDDGFDVDEMLRMMEADPHNEGGADAGMGQPWCLDGLDSSVLESMLQSEPEPFLMSEEPEMFLAGFESPSSFFEGLERLK*
A3	SbERF7 (Sb01g040280.1)	MVKNPGSNGVLTATAFSDNKLARPESGVVGNGKAAARPYKGVRMRSWGSWVSEIRAPNQKRRIWLGSYATPEAAARAYDAALLCLKGSDAVLNFPATSTSSSASASSSHRRADKDDDPAAGGGMSPRSIQRAAAAAAAAIDADAGGISADDRCSSSACAMTPTSASLSSTQGSSDHVRQEQHATTTSPAAASTGSPPEGEELWTDLEAFASPKFMDLVDTGAAAPFSSTWEEPEDDGELMRLWSFC

Continued Table S3

Subgroups	Protein name	Amino acid sequence
A3	SbERF40 (Sb03g042060.1)	MMRRAEPVGEAADAERRGRGGYKGVRRRRWGKWVSEIRVPGTRERLWLGSYATPEAAAVAHDTAVYFLRGGTGAGAGTGAGVAGGGDVAALNFPERAAAAYGTGAGAGAAGRLSPRSVQRVASDAGMAADAQLVAARESAPAPALAHAHRTGIGIGSAHDGGASAHARPGAGREQPAVSGEINVDDMEILM
A3	SbERF55 (Sb04g034290.1)	MVKSAQLQDTAVDGAAGNAVAARQQGAGAPAGYGSGGGKRQYKGVRMRSWGSWVSEIRAPNQKTRIWLGSYSTAEAAARAYDAALLCLKGSAADLNFPVHLPFHIPAAAMSPKSIQRVAAAAAANATCSPLPAAAAPYSASTAVNDGATPPCSYYGDASSGVSSPETGNADLCHDGMDMAGDADFAALADIEAFFQSPKCMEYGMMDPCSTFFAPAPMATDAANEWEEEGEINLWSFSSLN
A3	SbERF82 (Sb07g022265.1)	MDDGAGASRSDGEGSPRLPAERRYKGVRFRKWGRWVSEIRMPNSRERIWLGSYSSAEKAARAFDAAAVCLRGSRAGSLNFPESPPNVRHIPGAMLTPEQIQAEAARHANQLLPSPPVASPAASSSSQPAAAGGASSDRTTLSMPPSTYYGSGAAVCGDDEALDWSFMDALPSMPASSMGMGNSGADIVPALDDFMYGSPHPVMPPSEEVTQDMIDDDDDHTFISDDLWRF
A3	SbERF94 (Sb09g020690.1)	MSRAASGGGAGTGRGGGGGAERAHRCRYRGVRRRAWGKWVSEIRVPGTRERLWLGSYAAPEAAAVAHDAAACLLRGGGGRAAGQAHLNFPDRAACYIVYGGSHGHGPPLSPRSVQRVASDAGMAADAQIVDARAAAAALAAPTTRVQPAAFAGIGAAQGGAQGAACAPPPYSDGASSSSSSTYWSTPSASASRTSSSTGSEQPLVFGDISVDDIEILV
A3	SsAP2/ERF-056	MSSSPETEAGSSSGGKKFKGVRKRKWGKWVSEIRLPNSRERIWLGSYDAPDKAARAFDAAFVCLRGRGAAGADLNFPDSPPPCRAGGCSSDPREVQAAALSHANRAAVTAQQAAAAFIMDDAEADGGSAAPWDYYYSVAHDAGGVLGAAATSATEVVAPVRADGSIDWRPIMAHPPPLFSPTGWGSNAYDFLQVPPPAAAVADEDMDDGIHGATASLWSFDRDSYFRH
A3	SsAP2/ERF-071	MVKTAVAASSNDAAAARHGGSGKQRTYKGVRMRSWGSWVSEVRAPGQKTRIWLGSHSTADAAARAYDAALLCLKGSAAAPDLNFPLRLPFDLPPAGAMSPKAIQRVAAAAAASSATPFAPCAENNNGSACTDGDGDITPAWSSSSPARDDVSSPESTVSSESDLSGD
A3	SsAP2/ERF-097	MNDGAGASRSDGEGSPRLPAERRYKGVRFRKWGRWVSEIRMPNSRERIWLGSYSSAEKAARAFDAAAVCLRGSRAGSLNFPESPPNVRHIPGALLTPEQIQAEAARHANQQLPSPPVASPASSSSQPAPAPPAGGASSDRTALSMPSTYYSSGDAVCGDDEALDWSFMDALPSSMPASSVGMGNSADIVPALDDFMYGSPHQVMPPSEEATHDMIDSDDDHTFISDDLWRF*
A4	SbERF46 (Sb04g022943.1)	MENHHLQQQQQQQQPAAAATASSPQYRGVRRRKWGKWVSEIRQPGTKVRVWLGSFDSAEAAAVAHDVAALCLRGPRDAQLNFPGSAGWLPRPPSTDPADIRAAAAEAAERVRREPALVGTSAAAAAEPGAGSRVHAASASRLDLAVGDEFDDDLASPRLWTEMAEAMLLDPPKWGPDGSDGSDGSGSQHWPQGSLWDAC
A4	SsAP2/ERF-069	MATKQFRMAAAYSGTSMHEQPLRCITTRWRWPWRLLYKSAVITRESTLHLHHRSEASYLLSSSGRALPHSMENHQQQQQPAAAASSPQYRGVRRRKWGKWVSEIRQPGTKVRVWLGSFDSAEAAAVAHDVAALCLRGPRDAQLNFPGSAGWLPRPASTDPADIRAAAAEAAERVRREPALVGTAAAAAEPGSVPASASGRLDLAVGDEFDDDLASPRLWTEMAEAMLLDPPKWGPDGSDGSEGSGSQHWPQGSLWDGC*

Continued Table S3

Subgroups	Protein name	Amino acid sequence
A5	SbERF6 (Sb01g029065.1)	MAQELQLETSSSASATTTSSCTTSCCSSTVTDSSSSSPAAANAAPAPRKRQA<br>AEAEAEAEAEAEAEAVEEEEEGCAGKTTAPAAKKRKRSSEGKHPTYRGVR<br>MRAWGKWVSEIREPRKKSRIWLGTFPTAEMAARAHDVAALAIKGRAATA<br>YLNFPDLAGVLPRAASAAPKDVQAAAALAAAFTSPSSPSSEPGDDAGAPA<br>APCDAREEPAAAAKGGAAPEETAAEEREPVPLPPPVSQPGTPSSSGVEEER<br>QLFDLPDLLLDIRDGFGCFPPMWAPLDTDVEEVVNAELRLEEPLLWE
A5	SbERF14 (Sb01g049400.1)	MEREQEQEAGTAQQLLGRRVRADTRHPVYRGIRYRGGKWVSEIREPRKSN<br>RIWLGTYPAPEMAAAAYDAAALALRGAEAALNFPGAAMSRPAPASCSPD<br>DIRAAAAAAAAAAVIGRSHSPQVGGEAAAGGGCGASTWSSGAGAQGQVP<br>EHRAGDRRIVDEDDVFQVPRLLAGMAEGLMMSPPRLVGPATDGAVLLEE<br>DGSEDGVVSLWDHS
A5	SbERF33 (Sb03g002630.1)	MAMSELSSDTSSTSSYSSDPSTSTSPRAAAPCSAAAAVSGKKRPRSNGGGES<br>GSAPAYRGVRMRAWGKWVSEIREPRKKSRIWLGTFPCPEMAARAHDVAA<br>LSIKGARAVLNFPDLARHLPRPASLAPRDVQAAAARAALMHMHSDGAG<br>SGSGSGGSSSPSSSSSSSTRDAADTDGAAAHDEPEPEPEPQRQHERQPVEM<br>AAELVFDELAPLWVEDVVEFGGPPSDHTWTPCDGLDAAVGFLHPLPLLW<br>DY
A5	SbERF51 (Sb04g031960.1)	MAEQPLPRPHSPSTPAAVQVQVQPGPAAASHQAAPSSGSAPASPRSPSPLL<br>LLLQGGDGGTSSAGAAAASSVATAPSTTMATSSGEPSPRSSGKHAFYRGIR<br>CRSGKWVSEIREPRKARRIWLGTYPTAEMAAAAYDVAARALRGADAVLN<br>FPGAIASRAAPASASPADIRAAAAAAAAAAQLEYAQSSQGTAANHPPPA<br>AARQDHRWHHQQQQHGVMMSGGASATADAAASLYNTPQQQQQQQEI<br>GGDEFMDEEAIFEMPQLLRNMAAGMMMSPPRLSPDTSDESPDPSEAGESL<br>WSYHDP
A5	SbERF52 (Sb04g032940.1)	MAMDDSSSGSEPEPTSSSSVEAPASPTASSSDSASAAGSDSKKRRRTKDGH<br>HPTYRGVRMRAWGKWVSEIREPRKKSRIWLGTFATAEMAARAHDVAAL<br>AIKGRAAHLNFPELAGELPRPATAAPKDVQAAAALAAAADFPATPANA<br>AGADDDCGPDASAASDDVSASASPPPATTLDNPDDALFDLPDLLLDLRH<br>GASSCQLLSCAPSWYDDVCFSGAAAGAFRLEEPLLWEY
A5	SbERF53 (Sb04g032960.1)	MEADALLHAPTTSSSCSDSGGGSCVINGAQERLSKTGGKPHKNVKRKRTA<br>SSPPAAPGGVAVAPEATVDDDCGGGGGRQRKRGSAGTRHPTFRGVRMR<br>VWGKWVSEIREPRKKSRIWLGTFPTAEMAARAHDVAALAIKGRAAHLNF<br>PHLAHELPRPASTSPADIQAAAAKAAAAAAAAAADADVEQCESSSHHIAE<br>TPSSSSSSAANSEEVAAASNSEESVLLFDFDLPDLLLDLRDGLWSPIWEAAA<br>PAAAAEEYDGDSLSEPLLWAHDHCWMDAAAVPVQPD
A5	SbERF69 (Sb06g024530.1)	MDAPAQRAMAVDTGNAPTTSCSSSSSTSSSSVVNDAPQEVVPKNSCKGTN<br>KRKRASSPDSQEQEVEANGSTNNGHHQGEESSSCCCSTDDTEASVGDDKA<br>EEAAAATATRSSRSGYKHPSYRGVRRRSWGKWVSEIREPRKKSRIWLGTFP<br>TAEMAARAHDVAALAIKGRAAHLNFPDRAHELPRPASTSPADIQAAAA<br>QAAAGAADVQCDAPPSPSPSPSPSSSAELLLPSSPAADADASPEQAAAAT<br>MTTTHGDGGGGQGQGQGQESALFDLPDLLLDLRDGLWWPPVWPAAAM<br>AAEEYDGCDVVGMHDEPLLWAE

Continued Table S3

Subgroups	Protein name	Amino acid sequence
A5	SbERF70 (Sb06g024540.1)	MEHEQVVSPQVSEGCCTCSSSSFSNTTTTSGGGGSLNASSPSSSDDSGGGGNKGTKRPRRDLKHPTYRGVRMRTWGKWVSEIREPRKKSRIWLGTFDNPEMAARAHDAAAVAIKGRAAHLNFPDLAHELPRAASAAPKDVQAAAALAAATVVVAASPAVVPSSCGHDDADAETEDLPPPERAMPECESVNQAQLEQLGGDIDSGLGFTFLDVPDALLDFGHMLSPLPLPSYCGSPWDDIADDLCFEEPLLLWEH
A5	SbERF71 (Sb06g025890.1)	MSDRGGPASPRSGKHPFYRGIRSRSGKWVSEIREPRKTRRIWLGTFPTAEMAAVAYDVAARALRGADAALNFPHLAASRPAPASTSADDIRAAAAEAAASLLLQQEQQQQPADHHQPPARGGAPGRGIAPAEAGAAQQQTGGSSAAAWAQQEGGSGAGNPYFLDEEALFETPQYLRNMAAGMMMSPPRFGRNSSDDSPDHPSSSDAGDSLWSYRDP
A5	SsAP2/ERF-067	MEEERALSPVSDACTTCSSSGGTGPNSPIASSSQSLDDTSGGGAGGSSSRKRPRRELKHPTYRGVRMRAWGKWVSEIREPRKKSRIWLGTFDTPEMAARAHDVAALAIKGRAAHLNFPEMSHELPRAASAAPEDVRAAAALAAAMETRESVAPPASSDSIHGARNEDNEEPAPSSSSGNDADDSAQSEEPAAPPSEHSMLADGHILDLALFELPDVLLEFGFALPPPLTSSVAALGALTTKRGERDMRAIPQYPGTARQPGHTLGSLFASLSYPLQHQYVHLCSSRPE*
A5	SsAP2/ERF-075	MAVDTSNAPTTSCSSSSSTSSSSVVNDAPQEVVPKNSCKGTNKRKRTSSPDSQEVEANGSTNNGHRVEESSSCCCSTEASVGNKEAEEAAAATATRSRSGYKHPSYRGVRRRSWGKWVSEIREPRKKSRIWLGTFPTAEMAARAHDVAALAIKGRAAHLNFPERAHELPRPASTSPADIQAAAAQAAAAAEVQCDAPPSPAPSPSSSAELLPSSPAADADASPEQAAADCCCPEAAAAATTTTTHGGDGGGQVQETALFDLPDLLLDLRDGLWWPPVWPAAAMAAEEYDCCDVVGMHEPLLWAE*
A5	SsAP2/ERF-076	MLLPDETNKQPESNVGSVLQTDHRPSEKRKQRETLLGVVISSKLSGFTRSPSSHSPRHQVSPQLSEGCTCSSSNTSSLNASSPSSSDDSGSVKQGTKKRPRRDLKHPTWGKWVSEIREPRKKSRIWLGTFDDPESAARAHDAAAVAIKGRAAHLNFPDLAHELPRAASAAPKDVQAAAALAAATVAAAASPVVIPSCAHAHGHGDADTETEELPPPERAMPEECETENQAQLEQLGGDIDIGLGCTFLDVPDALLDFGYMLSPLPLSSYCGSPWDDIADDLCFDEPLLLWEH*
A6	ZmDBF1 (AAM80486.1)	MQFIQAQLHLQRNPGLGPRAQPMKPAVPVPPAPAPQRPVKLYRGVRQRHWGKWVAEIRLPRNRTRLWLGTFDTAEQAALAYDQAAYRLRGDAARLNFPDNAESRAPLDPAVDAKLQAICATIAAASSSKNSKAKSKAMPINASVLEAAAASPSNSSSDEGSGSGFGSDDEMSSSSPTPVVAPPVADMGQLDFSEVPWDEDESFVLRKYPSYEIDWDALLSN
A6	SbERF8 (Sb01g044410.1)	MAATTIDWHGRNAALYGVADRDSKELVRALAPPMQQAAPTISFAYPCSGVEQQSAAAAAGSFLGAGGSSGLLTPAQILQLQSRLQFLRRPAAGGATLAAVTTQQMKRQGVPQAPAPLTSRPAVSKLYRGVRQRHWGKWVAEIRLPRNRTRLWLGTFDTAEEAALAYDSAAFRLRGDSARLNFPELRRGGQHLGPPLHAAVDAKLHAICNGTTDVVVPLPQSQSQSQSQSQSHATTATATTTPSSLSSASPHVKSEPGCSGSESSFSADGDVSSTGSSDVVPEMQLLDFSEAPWDESESFHLRKYPSLEIDWDSILIS

Continued Table S3

Subgroups	Protein name	Amino acid sequence
A6	SbERF21 (Sb02g023230.1)	MAAAIDLSGEELMRALEPFIRDASSAPHGSSPLLHPHHQPLSPSSPFSFHHAAAYGGYPFAAAAAEGAGQLSPAQMQYIQARLHLQRQSQQSSVLGPRAQPMKASAAAAPTPPRPQKLYRGVRQRHWGKWVAEIRLPRNRTRLWLGTFDTAEEAALAYDQAAYRLRGDAARLNFPDNAASRGPLHASVDAKLQTLCQNIAASKKGAKKHASASAAAAAATSSSAPTSNCSSSPSSDDASSSCCLESAAESSCPSPSPSASPESSTVPEMQQLDFSEAPWDEAAGFALTKYPSYEIDWDSLLAAN
A6	SbERF49 (Sb04g027660.1)	MAAAIDMYKYYNAHQIASSSPSDEELAKALEPFITSASSSSPYHRYSSSPSMSQDSYMPTPSYTSFATSPLPTPAATSSSSSPFSQLPPLYSSPYAAPSMVGQMGLNQLGPAQIQQIQAQFMFQQQQQQRGLHAAFLGPRAQPMKQSGSPPLAPAQSKLYRGVRQRHWGKWVAEIRLPKNRTRLWLGTFDTAEDAALAYDKAAFRLRGDMARLNFPALRRDGAHLAGPLHASVDAKLTAICQSLTGSKNGGSSGDESAGSPPDSPKCSASASTEGEEESGSAGSPPSPTLAPPVPEMAKLDFTEAPWDETETFHLRKYPSWEIDWDSILS
A6	SbERF80 (Sb07g020090.1)	MAAAINLPGPSEDLMRAMESFMQEDAAPSPLPMPPAAQYPPATPTHLSPAQMQFIQAQLHLQRNPGLGPRAQPMKPAVPIPPAQQRPAQKLYRGVRQRHWGKWVAEIRLPRNRTRLWLGTFDTAEEAALAYDQAAYRLRGDAARLNFPDNAASRAPLDPAVDAKLQAICATIAAASSKGGARAKQSKAMPINAPVLEATAACPSNSSSDEGSGSDEEMSSRTTTTPVVVAPPVVADMGQLDFSEAPWDEAESFVLRKYPSYEIDWDALLSN
A6	SbERF98 (Sb09g029070.1)	MDASLRTLPPAGSSFPGEVRSAVSSLLLSSPGGTSALDTVFSHLPPPVTIPPLGSSVYYRQCELLRHFAASQTHQPATATAAACSSSSSSSSAASASFQFQPQAPPDDAAAAAAMLRQKLYRGVRQRQWGKWVAEIRLPQNRVRVWLGTYDSPETAAHAYDRAAYRLRGEYARLNFPGVMDAPDTDCPDHLRQLRAAVDAKIQAIRARMARKRARARKQREEKESARSGSGANSSKPAAARPVASEGAATTTTTTTTSETSTTPYGSPDGVLSVSAVSAEGDCPLERMPSFDPELIWEMLNF
A6	SbERF103 (Sb10g007780.1)	MAAIDLYTNQLSSSSSSSSDQELMKALEPFIRSASSPTSSTSTTTSPFSYSYPYCSALPQDSYYLPATSSYTSFPPPPPAPTAATSFSQLPPLPQSSSSYASPAAASYPTSSSADAASGLAALNHLGPAAQIHQIQAQLLAQHQEQQQQQRGLLAAAAFLGPRATQPMKHAGAPSASSAKLFRGVRQRHWGKWVAEIRLPRNRTRLWLGTFDSAEDAALAYDKAAFRLRGDAARLNFPSLRRGGVHLAGPLDASVDAKLTAICQGLTTAEPASSKAADTDASTTATAAPDSPKASASTTTTEGDESVHSAGSPPSSLPAFFQPPPPPQQQHPEMVSLDFTEAPWDESAALHLNKYPSWEIDWDSILS
A6	SsAP2/ERF-004	MAATTIDWHGSNAALPAALYGVADSKELVRALAPPMEQAAPTISFSYPCSGVEHQSAAGSFLGAGGGSGLLTLAQILQVQSRLQFLRRPAAAGGGALAAVATQQMKRQGVPHAPAPAPLPARPAVSKLYRGVRQRHWGKWVAEIRLPRNRTRLWLGTFDTAEEAALAYDSAAFRLRGDSARLNFPELRRGGQHLGPPLHAAVDAKLHAICNGTLPQSQSNATAATATATTTTTPSSFSSASPHVKSEPGCSGSESSFSADGDVSSTGSSDV

Continued Table S3

Subgroups	Protein name	Amino acid sequence
A6	SsAP2/ERF-092	MAAAINLPGPSEDLMRAMESFMQDDAAPSPLAMPPPPRAPRLPL PAAGAHGHGHGAQQYPATTHLSPEQMQFIQAQLHLQRNPGLGP RAQPMKPAVPVPPQQRPQKLYRGVRQRHWGKWVAEIRLPRNRT RLWLGTFDTAEEAALAYDQAAYRLRGDAARLNFPDNAASRAPL DPAVDAKLQSICATIAAASSSSKGARAKSKAMPINAPVLEAPSNSS SDEGSGSGSGSGSDDEMCSSSATPVVVAPPVVADMGQLDFSEVPW DEAESFVLRKYPSYEIDWDALLSN*
A6	SsAP2/ERF-098	MAAAINLPGPSEDLMRAMESFMQDDAAPSPLAMPPPPPPRAPPL PLPAAGAHGHGHGAQQYPATTHLSPEQMQFIQAQLHLQRNPGL GPRAQPMKPAVPVPPQQRPQKLYRGVRQRHWGKWVAEIRLPRN RTRLWLGTFDTAEEAALAYDQAAYRLRGDAARLNFPDNAASRA PLDPAVDAKLQSICATIAAASSSSKGARAKSKAMPINAPVLEAPSN SSSDEGSGSGSGSGSDDEMCSSSATPVVAAPPVVTDMGQLDFSEVPW DEAESFVLRKYPSYEIDWDALLSN*
